# High‐Performance Nondoped Blue Delayed Fluorescence Organic Light‐Emitting Diodes Featuring Low Driving Voltage and High Brightness

**DOI:** 10.1002/advs.201902508

**Published:** 2019-11-27

**Authors:** Shi‐Jie Zou, Feng‐Ming Xie, Miao Xie, Yan‐Qing Li, Tao Cheng, Xiao‐Hong Zhang, Chun‐Sing Lee, Jian‐Xin Tang

**Affiliations:** ^1^ Jiangsu Key Laboratory for Carbon‐Based Functional Materials & Devices Institute of Functional Nano & Soft Materials (FUNSOM) Soochow University Suzhou Jiangsu 215123 China; ^2^ School of Physics and Electronics Science Ministry of Education Nanophotonics & Advanced Instrument Engineering Research Center East China Normal University Shanghai 200062 China; ^3^ Center of Super‐Diamond and Advanced Film (COSADF) Department of Chemistry City University of Hong Kong Hong Kong SAR China; ^4^ Institute of Organic Optoelectronics (IOO) JITRI Wujiang Suzhou 215215 China

**Keywords:** blue emission, nondoped TADF emitters, organic light‐emitting diodes, thermally activated delayed fluorescence

## Abstract

Thermally activated delayed fluorescence (TADF) provides great potential for the realization of efficient and stable organic light‐emitting diodes (OLEDs). However, it is still challenging for blue TADF emitters to simultaneously achieve high efficiency, high brightness, and low Commission Internationale de l'Eclairage (CIE) *y* coordinate (CIE*y*) value. Here, the design and synthesis of two new benzonitrile‐based TADF emitters (namely 2,6‐di(9*H*‐carbazol‐9‐yl)‐3,5‐bis(3,6‐diphenyl‐9*H*‐carbazol‐9‐yl)benzonitrile (2PhCz2CzBn) and 2,6‐di(9*H*‐carbazol‐9‐yl)‐3,5‐bis(3,6‐di‐*tert*‐butyl‐9*H*‐carbazol‐9‐yl)benzonitrile (2tCz2CzBn)) with a symmetrical and rigid heterodonor configuration are reported. The TADF OLEDs doped with both the emitters can achieve a high external quantum efficiency (EQE) over 20% and narrowband blue emission of 464 nm with a CIE*y* < 0.2. Moreover, the incorporation of a terminal *tert*‐butyl group can weaken the intermolecular π–π stacking in the nondoped TADF emitter, and thus significantly suppress self‐aggregation‐caused emission quenching for enhanced delayed fluorescence. A peak EQE of 21.6% is realized in the 2tCz2CzBn‐based nondoped device with an extremely low turn‐on voltage of 2.7 V, high color stability, a high brightness over 20 000 cd m^−2^, a narrow full‐width at half‐maximum of 70 nm, and CIE color coordinates of (0.167, 0.248).

## Graphical abstract

A new blue thermally activated delayed fluorescence emitter of 2tCz2CzBn is synthesized with a symmetrical and rigid heterodonor configuration, enabling significant suppression of self‐aggregation‐caused emission quenching. High‐performance nondoped organic light‐emitting diodes are achieved with a high external quantum efficiency of 21.6%, an extremely low turn‐on voltage of 2.7 V, and narrowband blue emission.

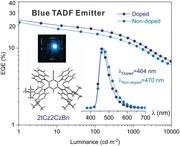

## 1 Introduction

Organic light‐emitting diodes are becoming the leading technology for full‐color displays due to their desirable features such as high efficiency, wide color gamut, light weight, and superior mechanical flexibility.^1^, ^2^, ^3^, ^4^, ^5^, ^6^, ^7^ Since the pioneer work by Adachi and co‐workers,^7^ thermally activated delayed fluorescence (TADF) materials without using heavy metals have gained great attention as the third‐generation organic light‐emitting diode (OLED) emitters.^8^, ^9^, ^10^, ^11^, ^12^, ^13^, ^14^, ^15^, ^16^, ^17^, ^18^, ^19^, ^20^ The use of pure organic TADF emitters allows almost 100% internal electroluminescence (EL) quantum efficiency by harvesting the triplet excitons (T_1_) to the emissive singlet excited states (S_1_) via reverse intersystem crossing (RISC). To date, high external quantum efficiency (EQE) exceeding 20% has been achieved for blue, green, and red TADF emitters in OLEDs.^8^, ^9^, ^10^, ^11^, ^12^, ^13^, ^14^, ^15^, ^16^, ^17^, ^18^, ^19^, ^20^ However, achieving high‐performance deep blue TADF OLEDs is still challenging, since most of them suffer from poor color purity, low brightness, severe EL efficiency roll‐off, as well as short operational stability.^21^, ^22^, ^23^, ^24^, ^25^ It remains an urgent demand to develop new blue TADF emitters that can simultaneously achieve high efficiency, high brightness, low driving voltage, long operational lifetime, and low Commission Internationale de l'Eclairage (CIE) *y* coordinate (CIE*y*) for various applications.

Several strategies have been proposed to realize highly efficient blue TADF emitters. Generally, the TADF molecules are designed with a donor–acceptor‐type rigid and symmetrical molecular configuration, exhibiting high optical bandgap (*E*_g_), small S_1_–T_1_ energy splitting (Δ*E*_ST_), and high photoluminescence quantum yield (PLQY).^26^, ^27^, ^28^ For narrowband emission, the multiple resonance effect has been explored to localize the frontier molecular orbitals on different atoms, which can minimize vibronic coupling between S_1_ and ground state (S_0_) to achieve the deep‐blue emission with a full‐width at half‐maximum (FWHM) of 18 nm.^28^, ^29^ Recently, efficient and stable blue TADF emitters have been successfully constructed by adopting the benzonitrile as a building block with the combination of two different donor units.^30^ Compared to the homodonor configuration,^14^ the heterodonor structures can enhance the RISC rate (*k*_RISC_) and shorten the delayed fluorescence lifetime, giving rise to the reduced efficiency roll‐off and the improved device stability. However, the sky‐blue emission with CIE*y* > 0.3 is inadequate for the display applications.^14^, ^30^, ^31^ Meanwhile, most reported TADF emitters exhibit broad EL spectra with a FWHM of 80–100 nm or even wider, leading to low color purity and significant energy losses in OLED displays with the use of color filters.^32^ In addition, most of high‐efficiency blue TADF emitters reported to date can only function well in the doped thin films, and encounter the serious performance degradation in nondoped devices due to the aggregation‐caused emission quenching (ACQ).^5^, ^8^, ^15^, ^16^, ^17^, ^18^, ^19^, ^20^, ^21^, ^22^, ^23^, ^24^, ^25^, ^26^ Therefore, it is highly desirable to further optimize molecular structures of blue TADF emitters to ameliorate the TADF behaviors in both doped and nondoped OLEDs.

Here, we report the design and synthesis of two new blue benzonitrile‐based TADF emitters with the use of heterodonors, namely 2,6‐di(9*H*‐carbazol‐9‐yl)‐3,5‐bis(3,6‐diphenyl‐9*H*‐carbazol‐9‐yl)benzonitrile (2PhCz2CzBn) and 2,6‐di(9*H*‐carbazol‐9‐yl)‐3,5‐bis(3,6‐di‐*tert*‐butyl‐9*H*‐carbazol‐9‐yl)benzonitrile (2tCz2CzBn). Both the emitters can exhibit blue emission at 464 nm with a CIE*y* < 0.2 and an EQE over 20% in doped TADF devices. More impressively, the replacement of the terminal phenyl group in the 3,6‐diphenyl‐9*H*‐carbazol‐9‐yl (PhCz) with a *tert*‐butyl group in the 3,6‐di‐*tert*‐butyl‐9*H*‐carbazol‐9‐yl (tCz) can weaken the intermolecular π–π stacking in the solid state and significantly suppress the ACQ, leading to PLQY enhancement in the neat TADF emitter.^30^ The nondoped TADF OLED with 2tCz2CzBn achieves a peak EQE of 21.6% with an extremely low turn‐on voltage of 2.7 V, ultrahigh color stability, a high brightness over 20 000 cd m^−2^, and a narrowband emission at 470 nm. To the best of our knowledge, this device is among the most‐efficient and brightest OLEDs based on nondoped blue TADF emitters.

## 2 Results and Discussion

### 2.1 Molecular Design and Synthesis

**Figure 1:**
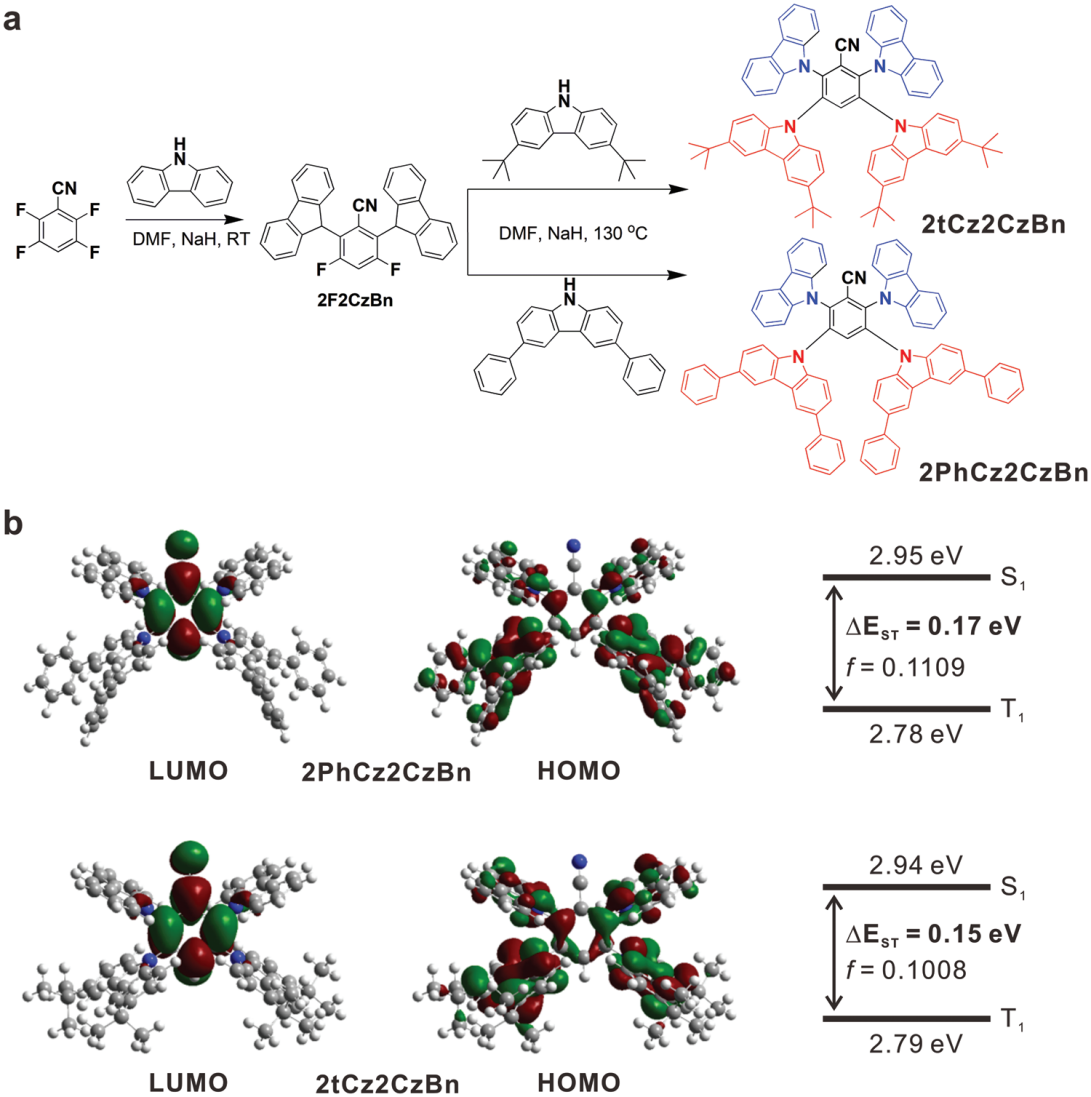
Synthetic scheme and theoretical calculations of blue TADF emitters. a) Synthetic schemes of 2PhCz2CzBn and 2tCz2CzBn. b) Calculated LUMO and HOMO distributions and energy levels of 2PhCz2CzBn and 2tCz2CzBn.

**Figure** **1**a depicts synthetic routes of two benzonitrile‐based blue TADF emitters, 2PhCz2CzBn and 2tCz2CzBn. The benzonitrile unit was selected as an acceptor, while the rigid and symmetrical PhCz and tCz units were used as a donor. Detailed synthesis procedures are described in the Supporting Information. Chemical structures of the synthesized materials were thoroughly characterized by ^1^H and ^13^C nuclear magnetic resonance (NMR), and mass spectrometry analysis (Figures S1–S6, Supporting Information).

Quantum‐chemical calculations were conducted to estimate geometric structures and electronic properties of 2PhCz2CzBn and 2tCz2CzBn by time‐dependent density functional theory (TD‐DFT) at B3LYP/6‐311G(d,p) level. Figure 1b illustrates the TD‐DFT calculation results, including the optimized S_0_ geometries of the two emitters in gas phase, the highest occupied molecular orbital (HOMO), the lowest unoccupied molecular orbital (LUMO), excited S_1_ and T_1_ levels, Δ*E*_ST_, and oscillator strength (*f*). To check the reliability of the exchange functionals in DFT calculations in case of charge‐transfer excitations and extended conjugated systems, the effects of different functionals on the molecular orbital and excitation energies were compared, as summarized in Figure S7 and Tables S1 and S2 (Supporting Information). It is thus confirmed that the calculated values under B3LYP functional are, to a large extent, reasonable and acceptable while comparing with the experimentally measured values. It is obviously shown in Figure 1b that both 2PhCz2CzBn and 2tCz2CzBn exhibit well separated HOMOs and LUMOs. The calculated LUMOs are entirely localized on the benzonitrile acceptor, while the HOMOs are mainly distributed on all four surrounding donors with slight extensions to the benzonitrile acceptor. As shown in Figure S8 (Supporting Information), due to their large steric hindrance, the PhCz and tCz donor units have large twisting angle of 56°–64° with the central accepting unit. These large twistings will break conjugation between the donors and the acceptors, leading to small HOMO–LUMO overlaps, reduced intramolecular charge transfer (ICT), small Δ*E*_ST_, and efficient RISC process.^33^ As shown in Figure 1b, 2PhCz2CzBn and 2tCz2CzBn exhibit small Δ*E*_ST_ values of 0.17 and 0.15 eV, respectively, together with strong *f* values of 0.1109 and 0.1008. The natural transition orbital analyses were also carried out to understand the S_0_ → S_1_ and S_0_ → T_1_ transition characters. Small hole–electron overlap integrals are present in the S_0_ → S_1_ transition due to the large twist angles between the donor and the acceptor, whereas the reduced donor–acceptor twist angles lead to the large hole–electron overlap integrals in the S_0_ → T_1_ transition (Figure S9, Supporting Information). As a result, the RISC processes in both 2PhCz2CzBn and 2tCz2CzBn can be enhanced due to the strengthened spin–orbit coupling.^34^

**Figure 2:**
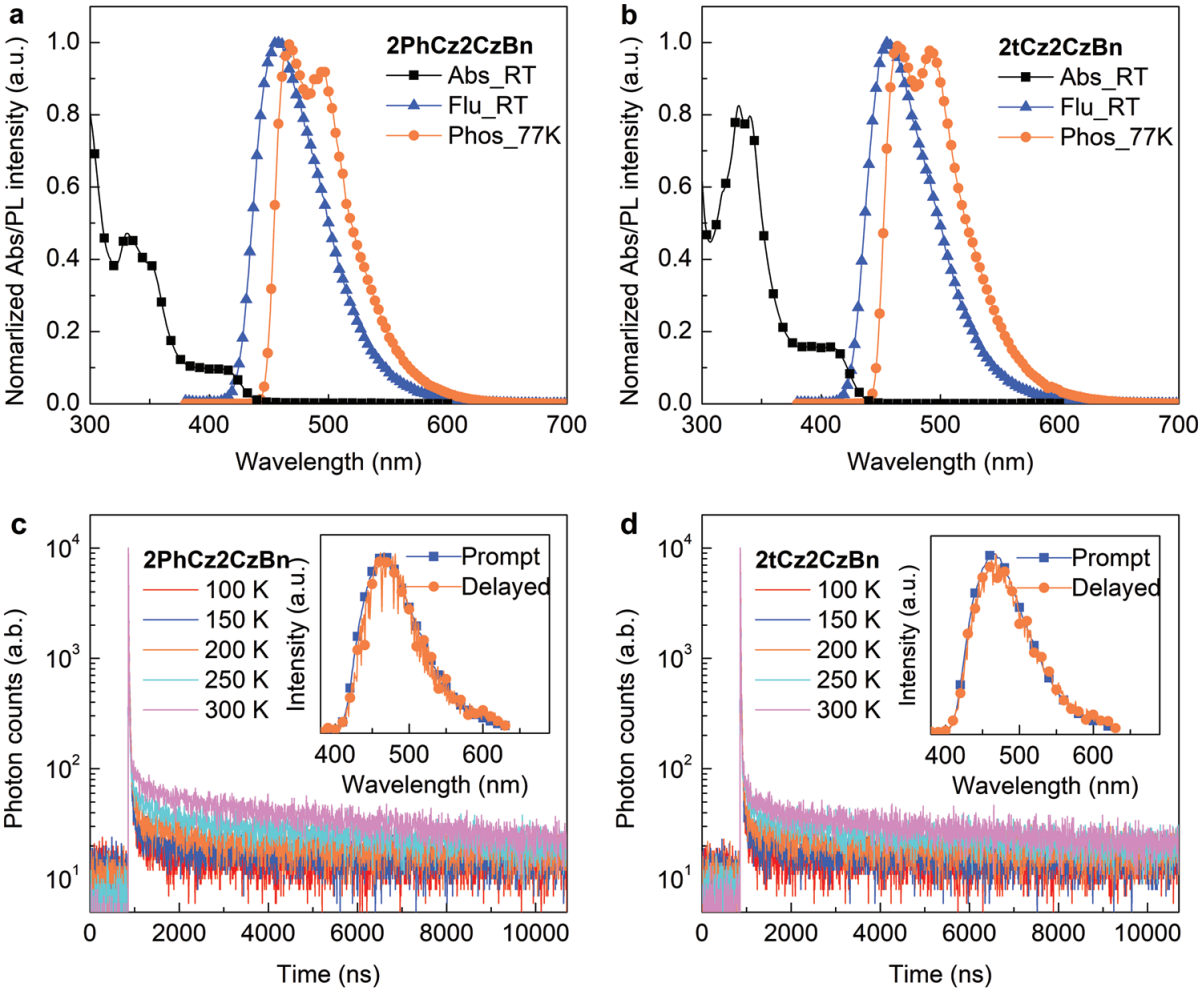
Photophysical properties of blue TADF emitters. a,b) Absorption spectra at room temperature in toluene, fluorescence spectra at room temperature, and phosphorescence spectra at 77 K in 2‐MeTHF for 2PhCz2CzBn (a) and 2tCz2CzBn (b). c,d) Temperature‐dependent transient PL decay profiles of 2PhCz2CzBn (20 wt%)‐doped films (c) and 2tCz2CzBn (30 wt%)‐doped films (d) with an mCBP host. Insets in (c) and (d) are the corresponding prompt (11 ns) and delayed (20 µs) spectra.

### 2.2 Photophysical, Electrochemical, and Thermal Properties

Photophysical properties of 2PhCz2CzBn and 2tCz2CzBn were examined by measuring the absorption characteristics in dilute toluene solutions at a concentration of 10^−5^ m and temperature‐dependent photoluminescence (PL) characteristics in 2‐methyltetrahydrofuran (2‐MeTHF) solutions and doped thin films with a 3,3′‐di(9*H*‐carbazol‐9‐yl)‐1,1′‐biphenyl (mCBP) host. **Figure** **2** displays the corresponding spectra, and **Table** **1** summarizes the detailed photophysical data. It is noteworthy that 2PhCz2CzBn and 2tCz2CzBn exhibit similar absorption and PL spectra (Figure 2a,b), in which the ICT‐induced absorption onsets occur at around 424 nm and their room‐temperature PL (fluorescence) peaks at 458 and 455 nm, respectively. *E*_g_ calculated from the absorption onset is ≈2.9 eV for both 2PhCz2CzBn and 2tCz2CzBn. The small overlaps between the absorption and PL spectra indicate strong charge transfer character in the excited states with the reduced self‐quenching and high PLQY. Particularly, both the emitters show narrowband blue emission with a FWHM of 64 nm. As determined from the onset wavelengths of room‐temperature fluorescence and low‐temperature phosphorescence spectra, the S_1_ and T_1_ values are determined to be 2.95 and 2.78 eV for 2PhCz2CzBn, and 2.94 and 2.79 eV for 2tCz2CzBn, respectively. From these energies, the Δ*E*_ST_ values are, respectively, estimated to be 0.17 and 0.15 eV for 2PhCz2CzBn and 2tCz2CzBn. The very small Δ*E*_ST_ values suggest that both the emitters can have high TADF performance. PLQYs of the 2PhCz2CzBn‐ and 2tCz2CzBn‐doped thin films with a mCBP host are found to be 86% and 87%, respectively (Table 1). Intriguingly, the PLQY of a nondoped 2PhCz2CzBn film sharply declines to 45%, whereas that of 2tCz2CzBn retains a value as high as 66%. These results indicate that ACQ behavior is more severe in the nondoped 2PhCz2CzBn film.^18^, ^35^ By contrast, 2tCz2CzBn shows much less concentration quenching in the solid state as observed to many TADF emitters.^35^, ^36^, ^37^, ^38^ In addition, the PL spectra of two emitters in solvents with various polarities reveal a significant bathochromic shift with the broadening in spectral shape (Figure S10, Supporting Information), indicating the presence of strong solvatochromic effect for both 2PhCz2CzBn and 2tCz2CzBn.

**Table 1:** Photophysical, electrochemical, and thermal properties of 2PhCz2CzBn and 2tCz2CzBn

Emitter	λ_abs_ ^a)^ [nm]	λ_Flu_ ^a)^ [nm]	FWHM^a)^ [nm]	λ_Phos_ ^b)^ [nm]	Δ*E*_ST_ ^c)^ [eV]	PLQY^d)^ [%]	τ_p_ ^e)^ [ns]	τ_d_ ^e)^ [µs]	*E*_g_ ^f)^ [eV]	HOMO^g)^ [eV]	LUMO^h)^ [eV]	*T*_d_ ^i)^ [°C]
2PhCz2CzBn	424	458	64	468	0.17	86^j)^/45^k)^	10.7	9.6	2.9	6.0	3.1	464
2tCz2CzBn	424	455	64	463	0.15	87^j)^/66^k)^	10.4	15.7	2.9	6.0	3.1	428

^a)^ Determined with 1 × 10^−5^ m at room temperature

^b)^ Determined with 1 × 10^−5^ m at 77 K

^c)^ Δ*E*_ST_ = S_1_–T_1_ determined from the onset wavelengths of fluorescence and phosphorescence

^d)^ Absolute PLQY by an integrating sphere

^e)^ Lifetime of the prompt and delayed components as determined from transient PL measurements

^f)^ Determined from the absorption onset at room temperature

^g)^ Determined by cyclic voltammetry with HOMO = (*E*_ox_ + 4.8) eV

^h)^ Determined LUMO = (HOMO − *E*_g_) eV

^i)^ Decomposition temperature determined by 5 wt% loss

^j)^ Determined from 20 wt% 2PhCz2CzBn‐doped and 30 wt% 2tCz2CzBn‐doped thin films with a mCBP host

^k)^ Determined from nondoped thin films.

To gain more insight into the delayed fluorescence behaviors, temperature‐dependent transient PL decay measurements were performed for 2PhCz2CzBn and 2tCz2CzBn doped into mCBP films from 100 to 300 K. The detailed rate constants, including radiative decay rate constant (*k*_r_), intersystem crossing (ISC) rate constant (*k*_ISC_), and RISC rate constant (*k*_RISC_) were also calculated according to the previous report,^8^ and summarized in Table 1 and Table S3 (Supporting Information). As shown in Figure 2c,d, both 2PhCz2CzBn and 2tCz2CzBn in doped thin films exhibit typical TADF features with nanosecond‐order prompt decays and microsecond‐order delayed decays. Moreover, the prompt and delayed PL spectra (insets of Figure 2c,d) are identical to each other, unambiguously confirming the presence of TADF behaviors for both the emitters. The delayed fluorescence emissions are even observed at 100 K for both the emitters, and the delayed fluorescent intensities are enhanced with increasing temperature. The calculated prompt lifetimes (τ_p_) at room temperature are 10.7 and 10.4 ns for 2PhCz2CzBn and 2tCz2CzBn, while the corresponding delayed lifetimes (τ_d_) are 9.6 and 15.7 µs, respectively. The fractional PLQYs for prompt/delayed fluorescence (Φ_p_/Φ_d_) at room temperature are calculated with the integral of the PL intensity ratios, which are 39%/47% for 2PhCz2CzBn and 41%/46% for 2tCz2CzBn, respectively (Table S3, Supporting Information). The *k*_RISC_ values of 2PhCz2CzBn and 2tCz2CzBn are calculated to be 2.20 × 10^5^ and 1.36 × 10^5^ s^−1^, respectively. The fast *k*_RISC_ and short delayed exciton lifetime of both the emitters arise from their small Δ*E*_ST_ values.

Electrochemical properties of 2PhCz2CzBn and 2tCz2CzBn were examined by cyclic voltammetry in dichloromethane solutions (see experimental details in the Supporting Information). The HOMO levels of 2PhCz2CzBn and 2tCz2CzBn are determined from the onset of the oxidation potentials, both of which are 6.0 eV (Figure S11, Supporting Information). By taking into account *E*_g_, the calculated LUMO levels are 3.1 eV for both the emitters (Table 1). Furthermore, thermal properties of 2PhCz2CzBn and 2tCz2CzBn were investigated by thermogravimetric analysis (TGA) and differential scanning calorimetry. According to the TGA measurements (Figure S12, Supporting Information), the decomposition temperatures (*T*_d_) at 5% weight loss are observed at 464 and 428 °C for 2PhCz2CzBn and 2tCz2CzBn (Table 1), respectively, indicating the excellent thermal stability. However, glass transition temperature (*T*_g_) was not observed for both the emitters.

### 2.3 Device Performance

**Figure 3:**
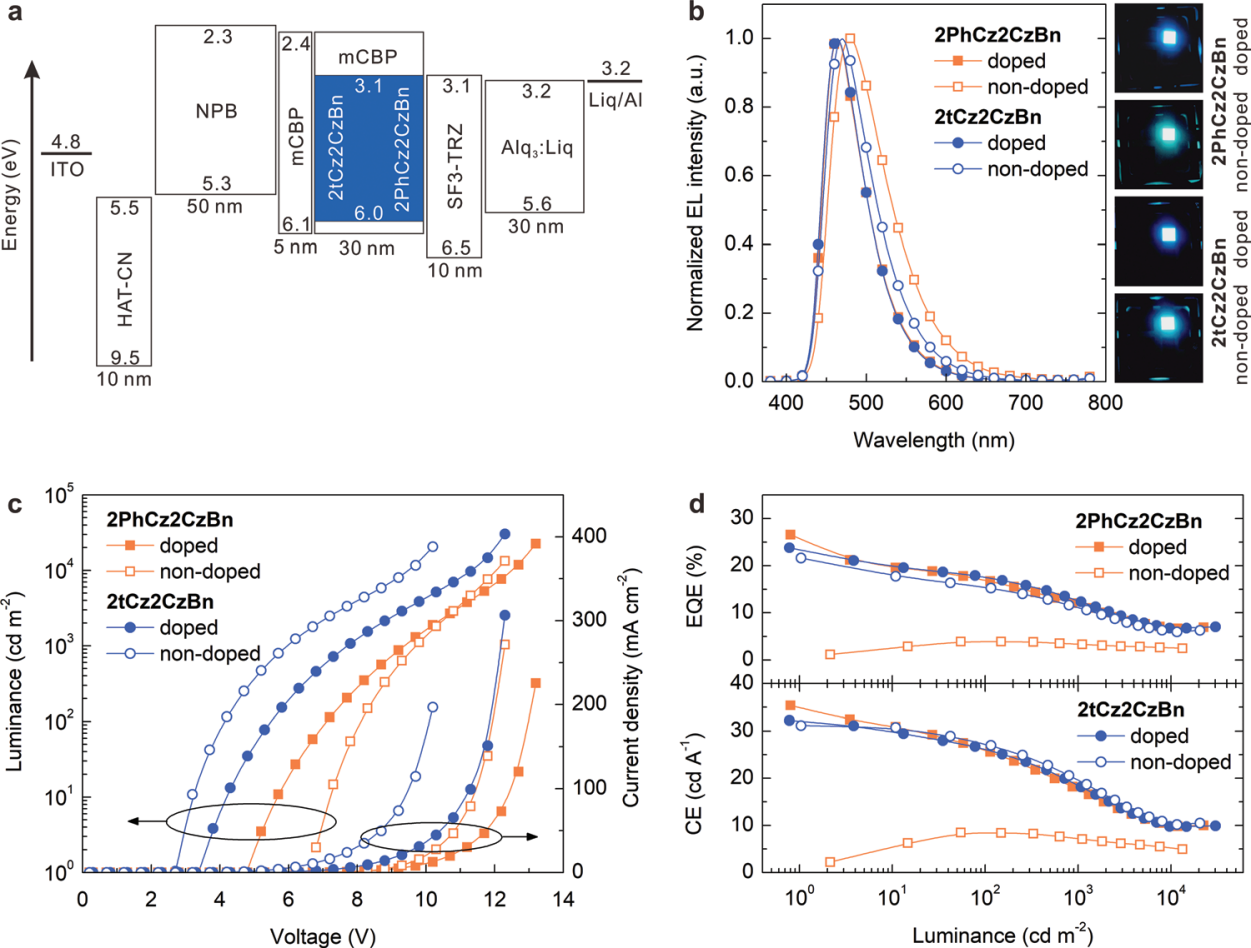
Performance characteristics of blue TADF OLEDs. a) Device structure and energy level diagram. b) EL spectra at 1000 cd m^−2^, and photos of the operating OLEDs. c) Current density and luminance as a function of driving voltage. d) EQE and CE as a function of luminance.

OLEDs with 2PhCz2CzBn and 2tCz2CzBn as the emitters were fabricated to evaluate their EL performance. **Figure** **3**a illustrates the device structure and a corresponding energy level diagram. The multilayered device architecture consists of glass/indium‐tin‐oxide (ITO) (120 nm)/2,3,6,7,10,11‐hexacyano‐1,4,5,8,9,12‐hexaazatriphenylene (HAT‐CN) (10 nm)/*N*,*N′*‐di(naphthalene‐1‐yl)‐*N*,*N′*‐diphenyl‐benzidine (NPB) (50 nm)/mCBP (5 nm)/TADF emitter‐doped mCBP or nondoped TADF emitter (30 nm)/2‐(9,9′‐spirobi[fluoren]‐6‐yl)‐4,6‐diphenyl‐1,3,5‐triazine (SF3‐TRZ) (10 nm)/8‐quinolinolato lithium (Liq) (30 wt%)‐doped tris(8‐hydroxyquinoline) aluminum (Alq_3_) (30 nm)/Liq (2 nm)/aluminum (Al) (100 nm). Here, ITO was used as an anode, HAT‐CN was used as a hole‐injection layer, NPB was used as a hole‐transport layer, mCBP was used as an electron‐/exciton‐blocking layer, 2PhCz2CzBn or 2tCz2CzBn doped in mCBP was used as an emitting layer, SF3‐TRZ was used as a hole‐blocking layer, Liq‐doped Alq_3_ was used as an electron‐transport layer, Liq was used as an electron injection layer, and Al was used as a cathode, respectively. The energy level diagram (Figure 3a) indicates that the electrons and holes can be effectively confined in the mCBP host for exciton formation in both 2PhCz2CzBn and 2tCz2CzBn due to the shallow LUMO level of mCBP and the deep HOMO level of SF3‐TRZ. The detailed device characteristics are presented in Figure 3b–d, and key performance parameters are listed in **Table** **2**.

**Table 2:** Device performance of blue OLEDs based on 2PhCz2CzBn and 2tCz2CzBn

Emitter	*V*_on_ ^a)^ [V]	λ_EL_ ^b)^ [nm]	FWHM^b)^ [nm]	CIE(*x*, *y*)^b)^	*L*_max_ ^c)^ [cd m^−2^]	EQE^d)^ [%]	CE^d)^ [cd A^−1^]
2PhCz2CzBN 20 wt% doped	4.7	464	61	(0.154, 0.200)	22 510	26.6/17.2/11.7	35.4/26.1/17.3
2PhCz2CzBN nondoped	6.7	480	84	(0.195, 0.333)	13 330	3.9/3.9/3.3	8.5/8.5/7.1
2tCz2CzBN 30 wt% doped	3.4	464	60	(0.153, 0.193)	30 290	23.8/17.1/12.4	32.2/26.0/18.2
2tCz2CzBN nondoped	2.7	470	70	(0.167, 0.248)	20 590	21.6/15.3/10.8	31.1/27.1/18.9

^a)^ Turn‐on voltage (*V*_on_) at 1 cd m^−2^

^b)^ EL peak, full‐width at half‐maximum (FWHM), and CIE coordinates recorded at 1000 cd m^−2^

^c)^ Maximum luminance

^d)^ External quantum efficiency (EQE) and current efficiency (CE) at their maximum, 100 and 1000 cd m^−2^.

Normalized EL spectra of TADF OLEDs based on the optimal doped emitters of 2PhCz2CzBn (20 wt%) and 2tCz2CzBn (30 wt%) exhibit the almost identical blue emissions with peak maxima at 464 nm and narrow FWHMs of 61 and 60 nm, respectively (Figure 3b). The corresponding CIE chromaticity (*x*, *y*) coordinates of 2PhCz2CzBn‐ and 2tCz2CzBn‐doped OLEDs are (0.154, 0.200) and (0.153, 0.193), respectively. It is also found that the emission spectra of TADF OLEDs rely on the doping concentrations of both 2PhCz2CzBn and 2tCz2CzBn in the mCBP host. As shown in Figure S13 and Table S4 (Supporting Information), the EL peaks of 2PhCz2CzBn‐doped devices redshift from 462 to 480 nm at a luminance of 1000 cd m^−2^ when increasing doping concentrations from 10 to 100 wt%, which are accompanied with variation of CIE (*x*, *y*) coordinates from (0.153, 0.188) to (0.195, 0.333). For 2tCz2CzBn, the EL peak shifts from 460 to 470 nm and the CIE (*x*, *y*) coordinates vary from (0.151, 0.170) to (0.167, 0.248) with increase in doping concentration from 10 to 100 wt% (Figure S14 and Table S4, Supporting Information).

Moreover, current density–voltage–luminance (*J*–*V*–*L*) characteristics of TADF OLEDs are rather dependent on the doping concentrations of both the emitters, revealing a substantial decrease in driving voltage when doping concentrations (Figures S13 and S14, Supporting Information). The enhanced electrical properties indicate the better conductivities of 2PhCz2CzBn and 2tCz2CzBn than that of the neat mCBP host. The optimal doping concentrations are determined to be 20 wt% for 2PhCz2CzBn and 30 wt% for 2tCz2CzBn, respectively (Table S4, Supporting Information). The optimal TADF OLEDs with both the emitters exhibit similar EL performance, achieving the maximum EQE and current efficiency (CE) of 26.6% and 35.4 cd A^−1^ for 2PhCz2CzBn and the maximum EQE and CE of 23.8% and 32.2 cd A^−1^ for 2tCz2CzBn, respectively (Table 2). The comparable EL performances between 2PhCz2CzBn‐ and 2tCz2CzBn‐doped devices can be attributed to the almost same PLQYs and Δ*E*_ST_ values.

Regardless of the similar device efficiencies, Figure 3c indicates that both 2tCz2CzBn‐doped and nondoped devices exhibit a lower driving voltage than that of 2PhCz2CzBn. The turn‐on voltages (*V*_on_) are 3.4 and 2.7 V for 2tCz2CzBn‐doped and nondoped OLEDs, respectively, while these values are considerably increased to 4.7 and 6.7 V for 2PhCz2CzBn‐based devices. More interestingly, the device efficiency of 2tCz2CzBn‐based OLEDs is, to a large extent, insensitive to the doping concentration, which is obviously different from the case of 2PhCz2CzBn (Table S4, Supporting Information). The nondoped OLED with 2tCz2CzBn achieves a maximum luminance of 20590 cd m^−2^ and a maximum EQE of 21.6%, which are almost identical to that of the optimal doped devices. Meanwhile, the EL spectrum of the nondoped device with 2tCz2CzBn exhibits a narrow FWHM of 70 nm with a CIE coordinate of (0.167, 0.248) at 1000 cd m^−2^, which is more blue than that of (0.195, 0.333) for 2PhCz2CzBn‐based one (see photos in Figure 3b). The CIE coordinates of 2tCz2CzBn‐based nondoped device are also rather stable under various driving voltages, in which the CIE*x* remains almost steady along with a slight shift of CIE*y* (<0.02) to the blue side (Figure S15, Supporting Information). The stable emission spectra indicate the presence of a stable exciton recombination zone under different driving voltages. To our best knowledge, the results of 2tCz2CzBn‐based nondoped OLEDs are among the most‐efficient blue OLEDs based on nondoped TADF emitters with low driving voltage (≈2.7 V) and narrowband emission (CIE*y* < 0.3) (see the summary of the best reported blue and sky‐blue OLEDs based on TADF emitters in Table S5 of the Supporting Information). The doped devices based on either 2tCz2CzBn or 2PhCz2CzBn exhibit excellent operation stability, and their half‐lifetimes at an initial luminance of 500 cd m^−2^ are estimated to be 215 and 285 h, respectively (Figure S16, Supporting Information). However, nondoped devices have the shorter operational lifetimes (e.g., 18 and 16 h 2tCz2CzBn and 2PhCz2CzBn), which are ascribed to some nonradiative recombination channels induced by triplet–triplet annihilation and singlet–triplet annihilation.

**Figure 4:**
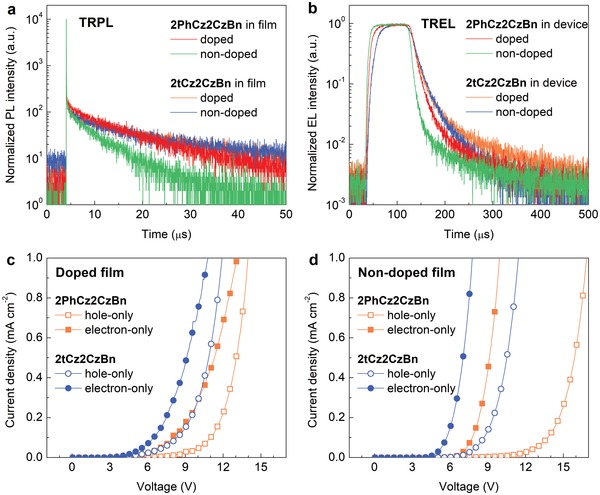
Dynamic emission and charge transport characteristics of blue TADF emitters. a) Transient PL and b) transient EL decay curves of 2PhCz2CzBn and 2tCz2CzBn in doped and nondoped films/devices with a mCBP host. c) Hole‐only and electron‐only devices with 20 wt% of 2PhCz2CzBn‐doped and 30 wt% of 2tCz2CzBn‐doped films. d) Hole‐only and electron‐only devices with 2PhCz2CzBn and 2tCz2CzBn nondoped films.

To better understand the carrier dynamics in nondoped OLEDs with 2tCz2CzBn, transient emission properties were measured for doped and nondoped thin films with both the emitters. The transient PL decay curves displayed in **Figure** **4**a indicate that the 2tCz2CzBn nondoped film exhibits delayed fluorescence characteristics similar to that of doped films, yielding a long delayed lifetime of τ_d_ = 9.8 µs and the fractional PLQYs of *Φ*_p_ = 31% and *Φ*_d_ = 35% (Table S3, Supporting Information). By contrast, the 2PhCz2CzBn nondoped film shows a remarkably shortened delayed lifetime of τ_d_ = 5.1 µs with a lower PLQY. The similar trends are also observed in transient EL characteristics (Figure 4b), in which the nondoped device with 2PhCz2CzBn exhibits the fastest EL decay along with the lowest device efficiency. Therefore, the high efficiency in nondoped devices with 2tCz2CzBn can be correlated to the improved delayed fluorescence as compared to the case of 2PhCz2CzBn. Considering the use of phenyl groups in the PhCz unit, a *tert*‐butyl group in the tCz unit is expected to reduce the intermolecular π–π stacking in the nondoped films, which can significantly suppress the ACQ for triplet excitons through intermolecular electron‐exchange interactions (i.e., triplet–triplet annihilation or triplet–polaron annihilation) and thus improve the PLQY and device efficiency.^18^, ^35^, ^39^, ^40^, ^41^, ^42^, ^43^

To elucidate the charge transport properties, hole‐only and electron‐only devices (HOD and EOD) were also fabricated. Here, the HOD has a structure of ITO/HAT‐CN (10 nm)/NPB (50 nm)/mCBP (5 nm)/doped TADF:mCBP or nondoped TADF emitter (30 nm)/mCBP (5 nm)/NPB (50 nm)/Al (100 nm), and the EOD uses a configuration of ITO/Alq_3_:Liq (30 nm, 30 wt%)/SF3‐TRZ (10 nm)/doped TADF:mCBP or nondoped TADF emitter (30 nm)/SF3‐TRZ (10 nm)/Alq_3_:Liq (30 nm, 30 wt%)/Liq (2 nm)/Al (100 nm). Figure 4c,d reveals that both HOD and EOD with doped and nondoped 2tCz2CzBn exhibit the higher electrical conductivities than those with 2PhCz2CzBn, which ascribe to the lower *V*_on_ for doped and nondoped devices with 2tCz2CzBn (Figure 3c). Meanwhile, the 2tCz2CzBn‐based devices show better bipolar charge transport ability than that of 2PhCz2CzBn. It has been demonstrated that the use of electron‐transport n‐type hosts in TADF OLEDs can broaden the exciton recombination zone by intrinsically balancing the charge fluxes.^24^ According to the energy diagram in Figure 3a, these well‐matched LUMO levels between 2tCz2CzBn and SF3‐TRZ are favorable for electron injection, resulting in the efficient exciton formation and radiative recombination in the nondoped emitting layer. This may be one of the determining factors for the high efficiency in nondoped OLEDs with 2tCz2CzBn.

## 3 Conclusion

In summary, two new benzonitrile‐based TADF emitters, 2PhCz2CzBn and 2tCz2CzBn, have been synthesized with a symmetrical and rigid heterodonor configuration, both of which exhibit small Δ*E*_ST_, fast *k*_RISC_ rate, and high PLQY. The TADF OLEDs doped with both the emitters can simultaneously achieve a high EQE over 20% and narrowband blue emission with an EL peak of 464 nm and CIE*y* < 0.2. More impressively, the use of a terminal *tert*‐butyl group in tCz can weaken the intermolecular π–π stacking in the solid state, and thus significantly suppress self‐aggregation‐caused emission quenching for improving PLQY in nondoped TADF emitters. A peak EQE of 21.6% is realized in the 2tCz2CzBn‐based nondoped blue device with an extremely low turn‐on voltage of 2.7 V, high brightness over 20 000 cd m^−2^, a narrow FWHM of 70 nm, and CIE coordinates of (0.167, 0.248). These results represent a substantive step toward the development of efficient blue TADF OLEDs.

## 4 Experimental Section

*Chemical Synthesis*: All the reagents were purchased from commercial sources and used without further purification. Detailed synthesis procedures of two emitters are described in the Supporting Information. All the reactions were performed under nitrogen atmosphere, and the crude products were purified by column chromatography before material characterizations and device fabrication.

*Measurements and Characterization*: Chemical structures were determined by ^1^H and ^13^C nuclear magnetic resonance spectra with a Bruker AVANCE III type NMR Spectrometer in CDCl_3_ solution, and mass spectra on Agilent 1260‐6125 (atmospheric‐pressure chemical ionization mode). Ultraviolet–visible absorption and PL spectra were recorded at room temperature with a Perkin‐Elmer Lambda 750 UV‐Vis spectrophotometer and a FM‐4 type fluorescence spectrophotometer (JY company, French), respectively. Optical bandgap (*E*_g_) was determined from the onset of the absorption spectra. Low‐temperature phosphorescence spectra were measured with a FLS 920 spectrometer (Edinburgh Corporation) in 2‐MeTHF at 77 K. Absolute PLQYs of doped and nondoped films on quartz substrates by vacuum evaporation were obtained with a C9920‐02G type fluorescence spectrophotometer (HAMAMASTU, Japan) with an integrating sphere at room temperature under nitrogen atmosphere. Transient PL decay curves of the films were measured with a Quantaurus‐Tau fluorescence lifetime spectrometer (C11367‐32, Hamamatsu Photonics) with an excitation wavelength of 373 nm, pulse width of 100 ps, and repetition rate of 5 kHz under vacuum atmosphere. Low‐temperature measurements were conducted using a cryostat (Oxford Optistat DN). Time‐resolved PL spectra including prompt and delayed components were measured by C11367 under vacuum. The time ranges of prompt and delayed parts were set at 11 ns and 20 µs with a wavelength range of 400–650 nm and a step of 1 nm.

*Theoretical Calculations*: All quantum chemical calculations were performed using a DFT method in Gaussian 09 program package. The ground‐state geometries were optimized by using a TD‐DFT approach at the B3LYP/6‐311G(d,p) level. The singlet and triplet excited‐state properties were calculated according to the optimized geometries.

*Device Fabrication and Measurements*: OLEDs were fabricated on patterned ITO‐coated glass substrates with a sheet resistance of ≈15 Ω sq^−1^. The ITO‐coated glass substrates were successively cleaned in ultrasonic baths with acetone, ethanol, and deionized water, and then dried in an oven at 110 °C. The ITO‐coated glass substrates were transferred into a high‐vacuum deposition chamber (base pressure ≤ 2 × 10^−6^ mbar) for the thermal deposition of organic materials and metal electrodes through a shadow mask. Layer thickness and deposition rate were in situ monitored by an oscillating quartz thickness monitor. Device active area was defined to be 0.1 cm^2^. After the film depositions, the fabricated devices were transferred to a connected nitrogen‐filled glovebox for the encapsulation with a glass cap and epoxy glue. Current density–voltage–luminance (*J*–*V*–*L*) characteristics and EL spectra of the devices were measured simultaneously with a source meter (Keithley model 2400) and a luminance meter/spectrometer (PhotoResearch PR670). The CIE 1931 color coordinates were obtained from the EL spectra. The EQE values were calculated by assuming an ideal Lambertian emission profile. The lifetime of the OLEDs was tested through a 64 channel ZJLS‐4 type OLED life aging testing system operated in the constant current mode at room temperature. Transient EL decay curves of the devices were measured under electrical excitation with a pulse width of 100 µs at 300 K generated by a pulse generator (Keysight 81150A).

## Conflict of Interest

The authors declare no conflict of interest.

## Supporting information

Supporting Information
