# Supplementary material for: High‐Performance Nondoped Blue Delayed Fluorescence Organic Light‐Emitting Diodes Featuring Low Driving Voltage and High Brightness
Source: Adv Sci (Weinh). 2019 Nov 27;7(3):1902508. doi: 10.1002/advs.201902508 (PMC7001635; doi:10.1002/advs.201902508)
Supplement: Supplementary file 1 — Supporting Information [file ADVS-7-1902508-s001.pdf]

Copyright WILEY-VCH Verlag GmbH & Co. KGaA, 69469 Weinheim, Germany, 2019.

ADVANCED SCIENCE Open Access

Supporting Information

for *Adv. Sci.,* DOI: 10.1002/advs.201902508

High-Performance Nondoped Blue Delayed Fluorescence Organic Light-Emitting Diodes Featuring Low Driving Voltage and High Brightness

Shi-Jie Zou, Feng-Ming Xie, Miao Xie, Yan-Qing Li, Tao Cheng, Xiao-Hong Zhang, Chun-Sing Lee,* and Jian-Xin Tang**

Copyright WILEY-VCH Verlag GmbH & Co. KGaA, 69469 Weinheim, Germany, 2019.

Supporting Information

High-Performance Non-doped Blue Delayed Fluorescence Organic Light-Emitting Diodes Featuring Low Driving Voltage and High Brightness

Shi-Jie Zou,[1,†] Feng-Ming Xie,[1,†] Miao Xie,[1,†] Yan-Qing Li,[1,2,*] Tao Cheng,[1] Xiao-Hong Zhang,[1] Chun-Sing Lee,[3,*] Jian-Xin Tang,[1,4,*]

[1] Jiangsu Key Laboratory for Carbon-Based Functional Materials & Devices, Institute of Functional Nano & Soft Materials (FUNSOM), Soochow University, Suzhou, Jiangsu 215123, China

[2] School of Physics and Electronics Science, Ministry of Education Nanophotonics & Advanced Instrument Engineering Research Center, East China Normal University, Shanghai, 200062, China

[3] Center of Super-Diamond and Advanced Film (COSADF), and Department of Chemistry, City University of Hong Kong, Hong Kong S.A.R., China

[4] Institute of Organic Optoelectronics (IOO), JITRI, Wujiang, Suzhou 215215, China

[†] These authors contributed equally to this work.

* Corresponding authors. E-mail: jxtang@suda.edu.cn (J.X. Tang); yqli@suda.edu.cn (Y.Q. Li); apcslee@cityu.edu.hk (C.S. Lee)

Keywords:

Thermally activated delayed fluorescence; organic light-emitting diodes; blue emission; non-doped TADF emitter

Supporting Notes

S1. Synthesis of 2,6-di(9H-carbazol-9-yl)-3,5-bis(3,6-di-tert-butyl-9H-carbazol-9-yl)benzonitrile (2tCz2CzBn).

3,6-Dophenylcarbazole (0.56 g, 1.75 mmol) was dissolved in dry dimethyl formamidine (DMF) in a three-neck flask under nitrogen atmosphere. The reaction mixture was cooled to 0 °C, and then NaH (0.04 g, 1.75 mmol) was added and stirred for 30 minutes. After that, **2F2CzBn** (0.40 g, 0.85 mmol) was added, and the reaction mixture was slowly warmed to room temperature. After 24 hours, the reaction was quenched with water, and the precipitate was filtered off. The crude product was purified by column chromatography to give 0.55 g (yield: 65%) of **2tCz2CzBn** as a pale-green solid. ^{1}H NMR (400 MHz, $CDCl_3$) δ 8.40 (s, 1H), 7.72 (d, J = 5.3 Hz, 8H), 7.26 (t, J = 8.3 Hz, 5H), 7.12 (dt, J = 13.6, 8.3 Hz, 17H), 1.34 (s, 36H). ^{13}C NMR (101 MHz, $CDCl_3$) δ 143.98, 139.06, 137.83, 137.32, 137.18, 136.65, 125.58, 124.37, 124.09, 123.21, 121.08, 120.08, 118.33, 116.06, 113.22, 110.05, 108.91, 77.37, 77.25, 77.05, 76.73, 34.62, 31.84. MS (APCI) calcd. for $C_{71}H_{65}N_5$: m/z = 987.52, found: 988.45 [M]+.

S2. Synthesis of 2,6-di(9H-carbazol-9-yl)-3,5-bis(3,6-diphenyl-9H-carbazol-9-yl)benzonitrile (2PhCz2CzBn).

2PhCz2CzBn was synthesized according to the same procedure described above for the synthesis of 2tCz2CzBn except for the use of 3,6-diphenyl-9H-carbazole **(2PhCz)** as the reactant. ^{1}H NMR (400 MHz, $CDCl_3$) δ 8.49 (s, 1H), 8.04 (s, 4H), 7.79 (d, J = 7.5 Hz, 4H), 7.60 (d, J = 7.5 Hz, 9H), 7.50 – 7.39 (m, 14H), 7.34 (t, J = 7.3 Hz, 6H), 7.22 (dd, J = 14.2, 6.8 Hz, 7H), 7.15 (t, J = 7.4 Hz, 4H). ^{13}C NMR (101 MHz, $CDCl_3$) δ 141.31, 139.21, 138.90, 137.88, 136.64, 134.65, 128.80, 127.20, 126.87, 125.86, 125.32, 124.63, 124.41, 121.42,

120.47, 118.79, 109.91, 109.66, 77.33, 77.21, 77.01, 76.69, -0.01. MS (APCI) calcd. for $C_{79}H_{49}N_5$: m/z = 1067.40, found: 1067.45 [M]+.

S3. Cyclic voltammetry measurements

Cyclic voltammetry (CV) measurements were conducted on a RST 3100 electrochemical analysis equipment at room temperature with a conventional three electrode configuration, consisting of a platinum disk working electrode, a platinum wire counter electrode, a Ag/AgCl reference electrode, and the supporting electrolyte of tetrabutylammonium hexafluorophosphate (0.1 M). The cyclic voltammograms were obtained at a scanning rate of 50 mV s^{-1} with a DCM solution.

S4. Characterization of thermal properties

Thermal gravimetric analysis (TGA) was carried out with a HCT-2 instrument at a heating rate of 10 °C min^{-1} under nitrogen atmosphere. Differential scanning calorimetry (DSC) analysis were performed on a Pyris Diamond DSC Thermal Analyzer under a nitrogen flow at a heating rate of 10 °C min^{-1}. However, the glass transition temperature (T_g) for each emitter was not observed from the DSC measurements.

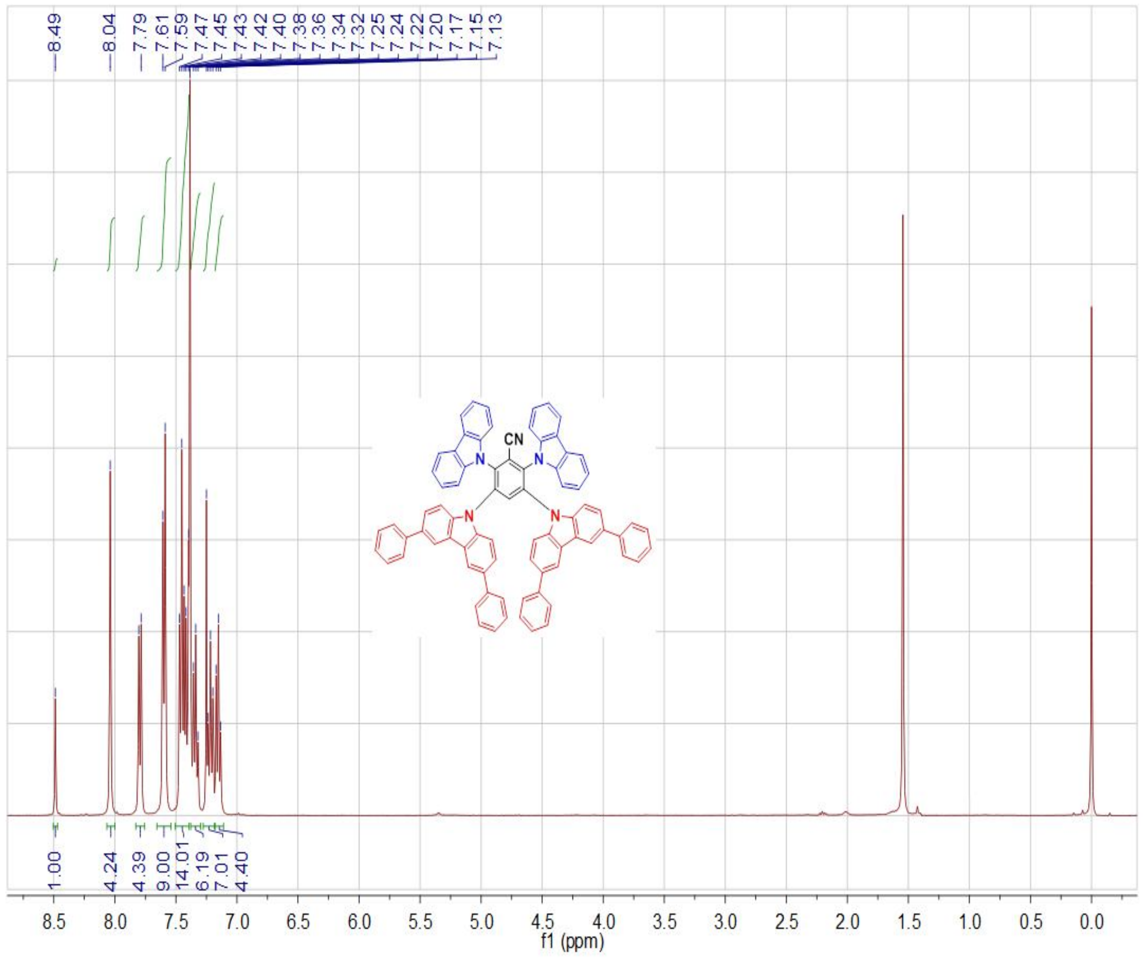

Figure S1. ^{1}H NMR spectrum of 2PhCz2CzBn.

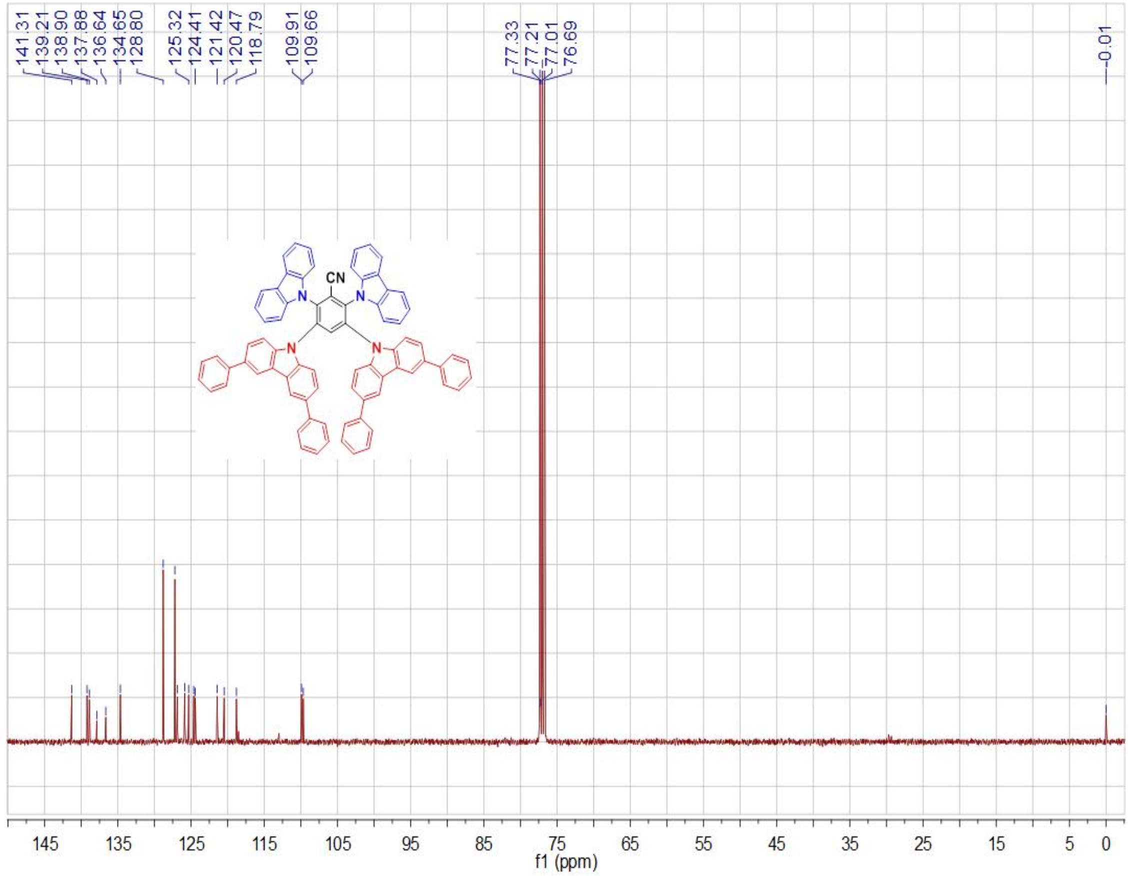

Figure S2. ^{13}C NMR spectrum of 2PhCz2CzBn.

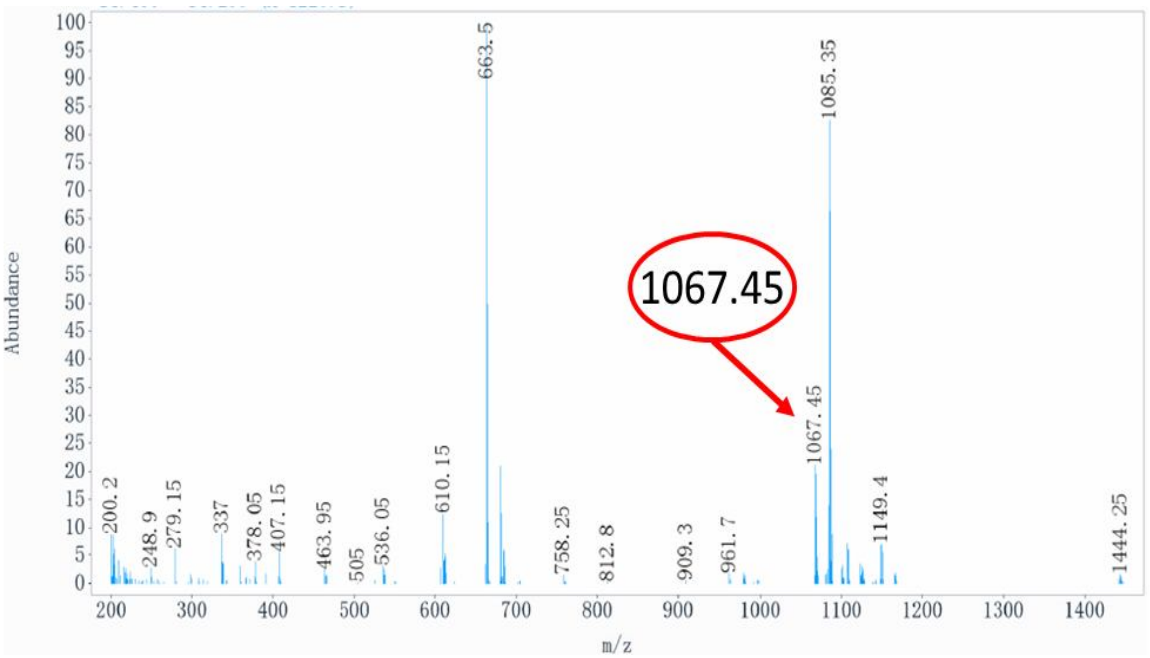

Figure S3. TOF-MS spectrum of 2PhCz2CzBn.

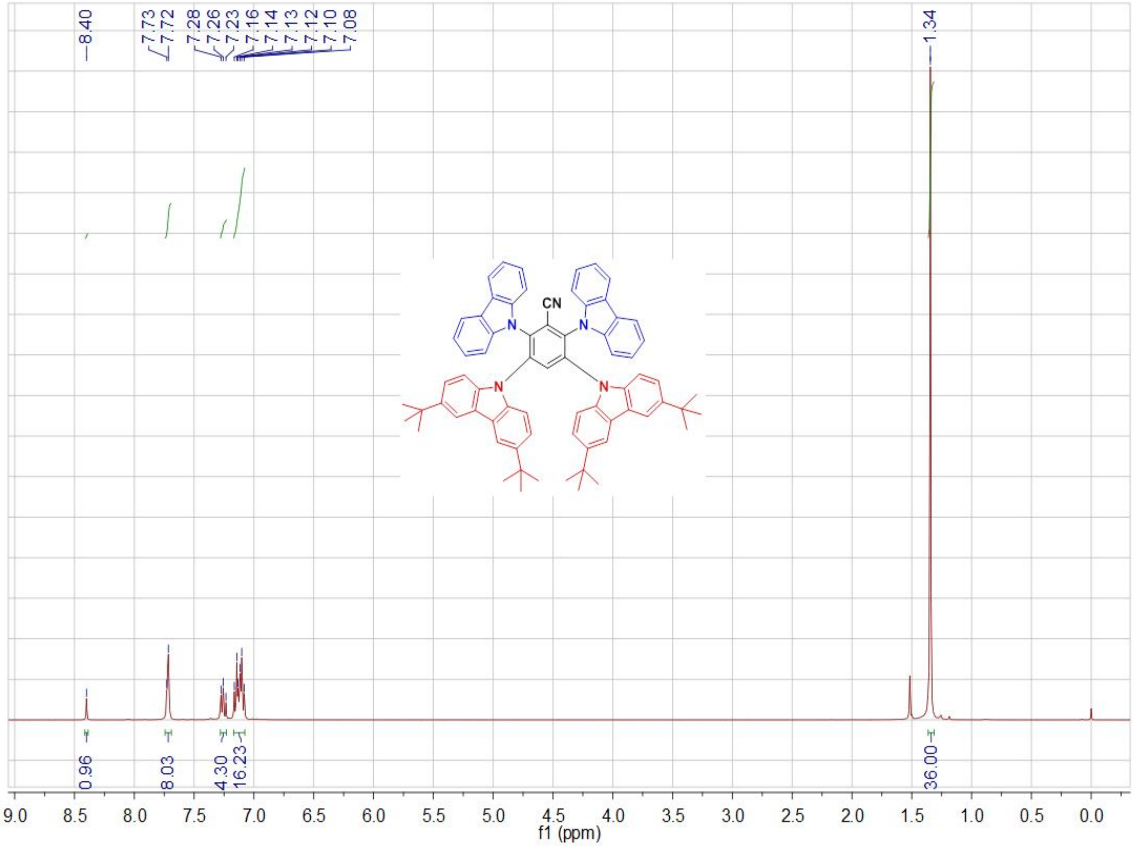

Figure S4. ^{1}H NMR spectrum of 2tCz2CzBn.

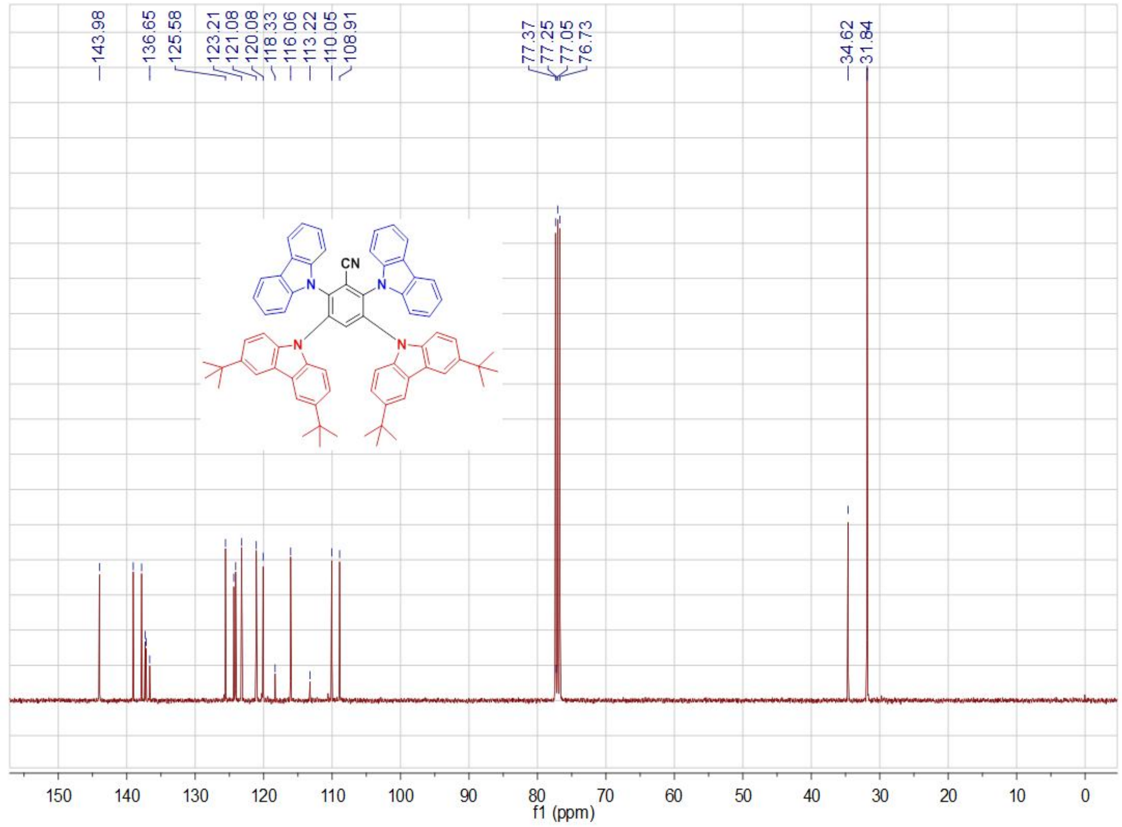

Figure S5. ^{13}C NMR spectrum of 2tCz2CzBn.

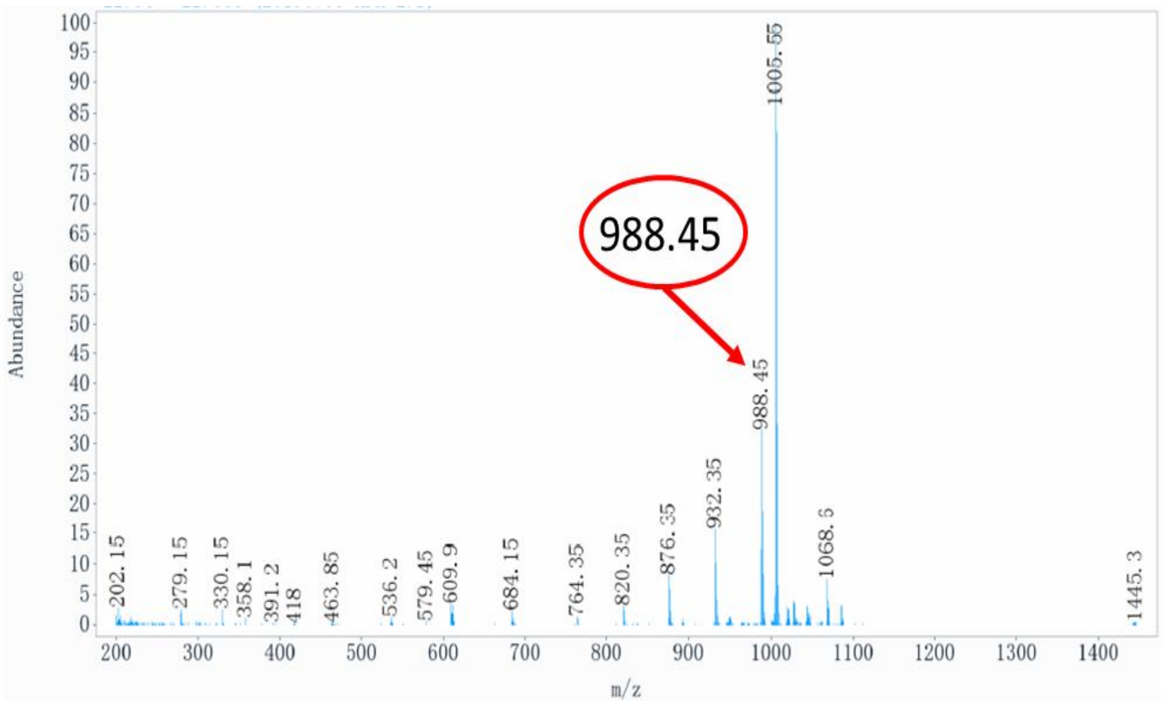

Figure S6. TOF-MS spectrum of 2tCz2CzBn.

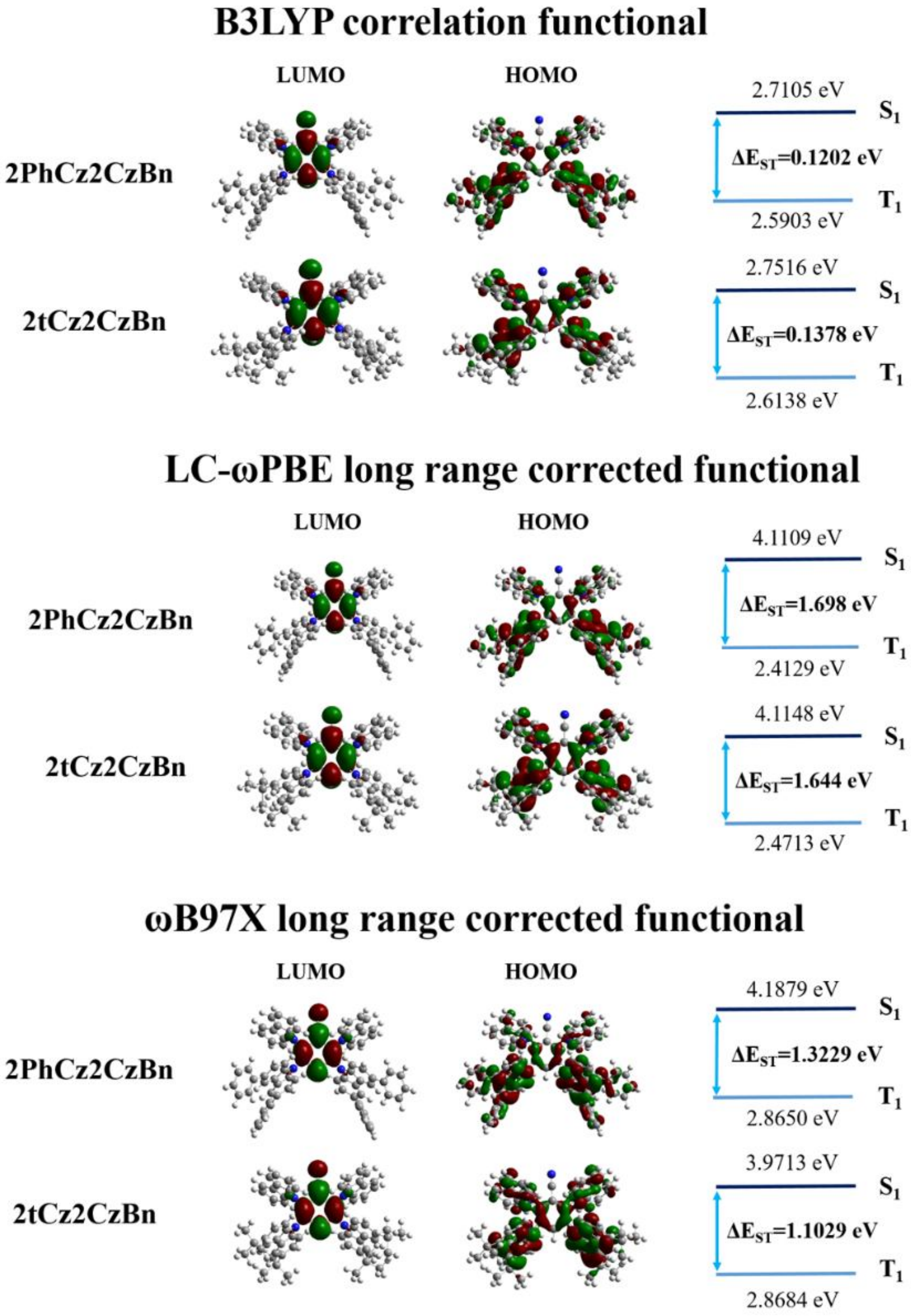

Figure S7. Calculated LUMO and HOMO distributions and energy levels of 2PhCz2CzBn and 2tCz2CzBn using different functionals.

Table S1. The effects of three functionals on the estimated vertical excitation energy with 6-31G(d) basic set in toluene solution.

Molecule	DFT functional			Exp
	B3LYP	LC-ωPBE	ωB97X	
	X [a] = 20%	0% → 100%	15.77% → 100% [b]	
2PhCz2CzBn	457 nm	301 nm	312 nm	424 nm
2tCz2CzBn	450 nm	301 nm	312 nm	424 nm

[a] X represents the percentage of nonlocal Hartree-Fock exchange.

[b] At short range → at long range for X value, respectively.

Table S2. Partial molecular orbital energies for all systems in toluene solution as given by different TDDFT calculations.

Materials	Level	B3LYP [eV]	LC-ωPBE [eV]	ωB97X [eV]	Exp [eV]
2PhCz2CzBn	HOMO	-2.163	-0.098	0.099	-3.1
	E_{L-H}	3.295	7.838	7.525	2.9
	LUMO	-5.458	-7.936	-7.624	-6.0
2tCz2CzBn	HOMO	-2.057	0.013	0.002	-3.1
	E_{L-H}	3.378	7.950	7.641	2.9
	LUMO	-5.435	-7.937	-7.639	-6.0

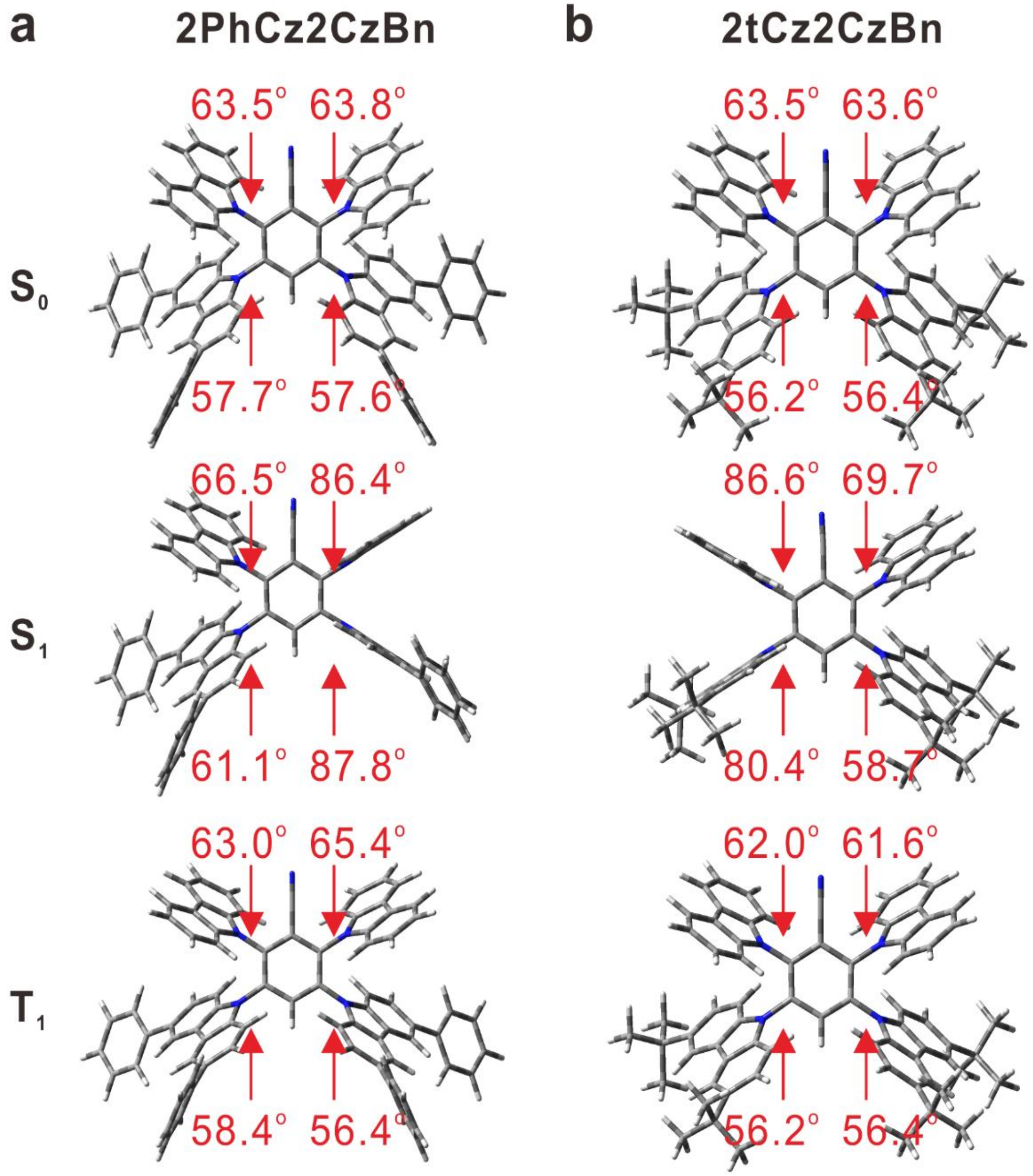

Figure S8. Dihedral angles between the donor and the acceptor in the optimized geometries at S_0, S_1, and T_1 states for a) 2PhCz2CzBn and b) 2tCz2CzBn.

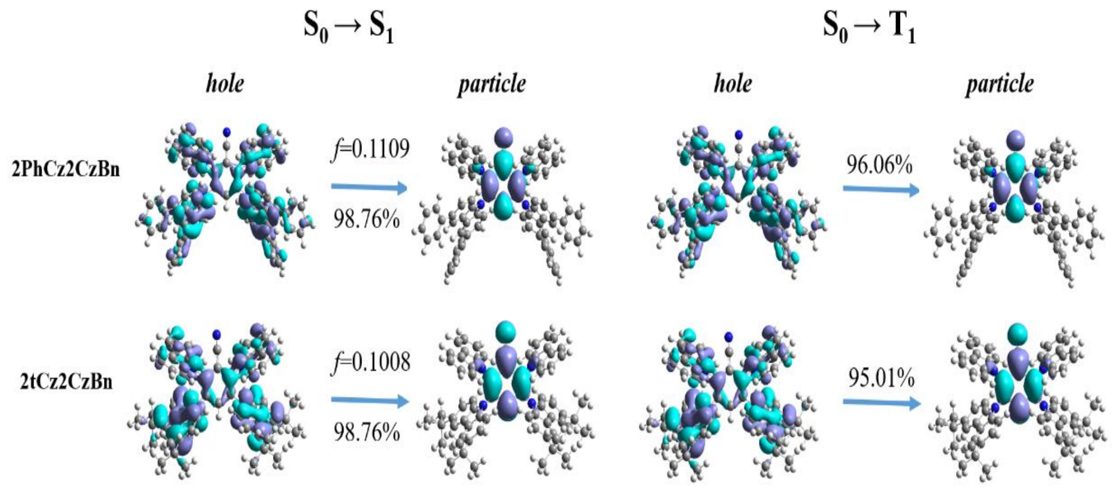

Figure S9. Natural transition orbital (NTO) analysis for 2PhCz2CzBn and 2tCz2CzBn.

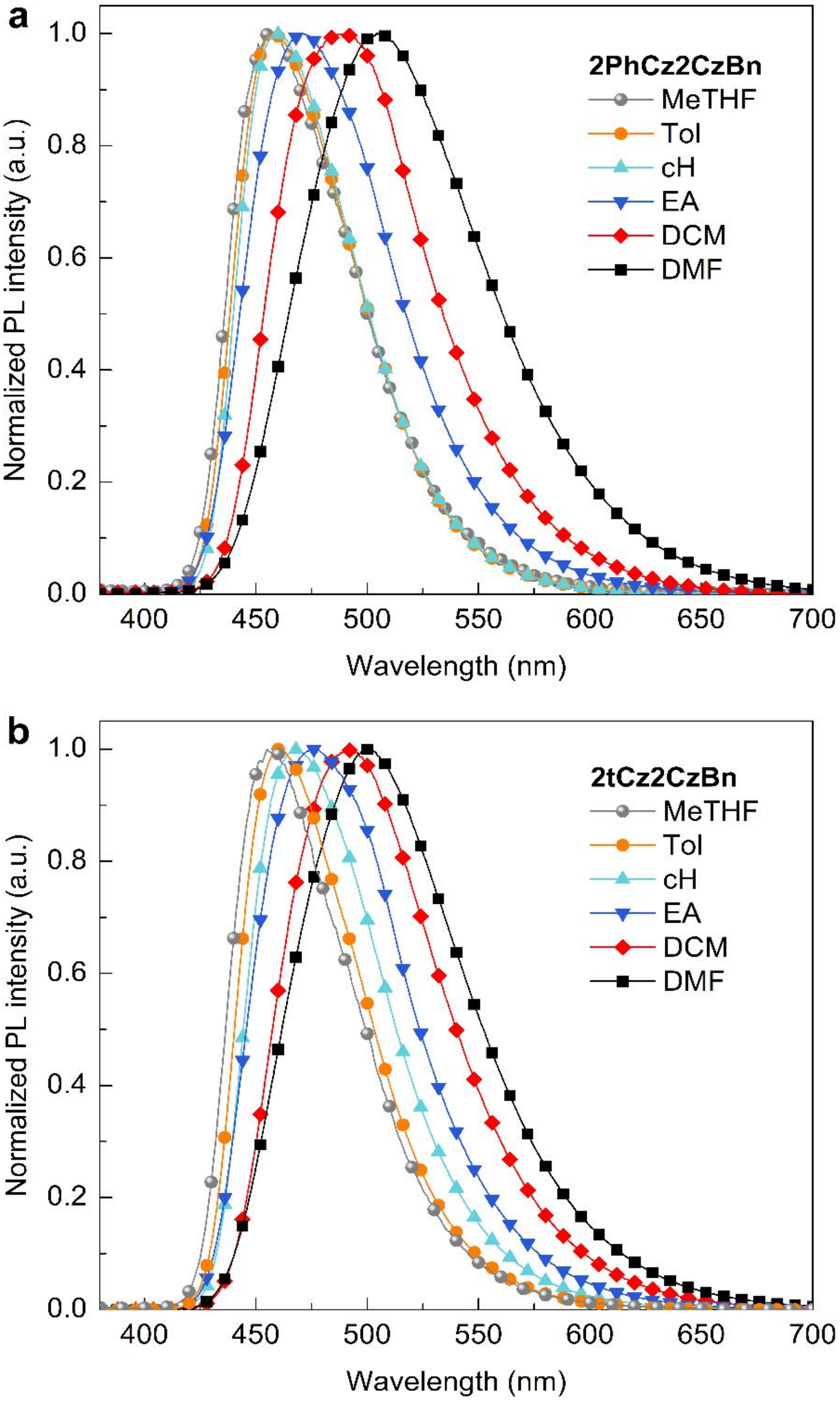

Figure S10. Normalized steady-state photoluminescence (PL) spectra of a) 2PhCz2CzBn and b) 2tCz2CzBn in various solvents with different polarity.

Table S3. Photophysical characteristics of 2PhCz2CzBn and 2tCz2CzBn in doped films with a mCBP host and non-doped films.

Films	τ_p [ns]	τ_d [μs]	τ_{EL} [μs]	PLQY/Φp/Φ_d [%]	k_r [10^7 s^{-1}]	k_{ISC} [10^7 s^{-1}]	k_{RISC} [10^5 s^{-1}]
2PhCz2CzBn 20 wt% doped	10.7	9.6	50.8	86/39/47	3.9	5.5	2.2
2PhCz2CzBn non-doped	17.4	5.1	36.0	45/25/20	2.3	4.2	3.4
2tCz2CzBn 30 wt% doped	10.4	15.7	62.1	87/41/46	4.1	5.2	1.4
2tCz2CzBn non-doped	15.7	9.8	52.3	66/31/35	2.0	3.4	2.1

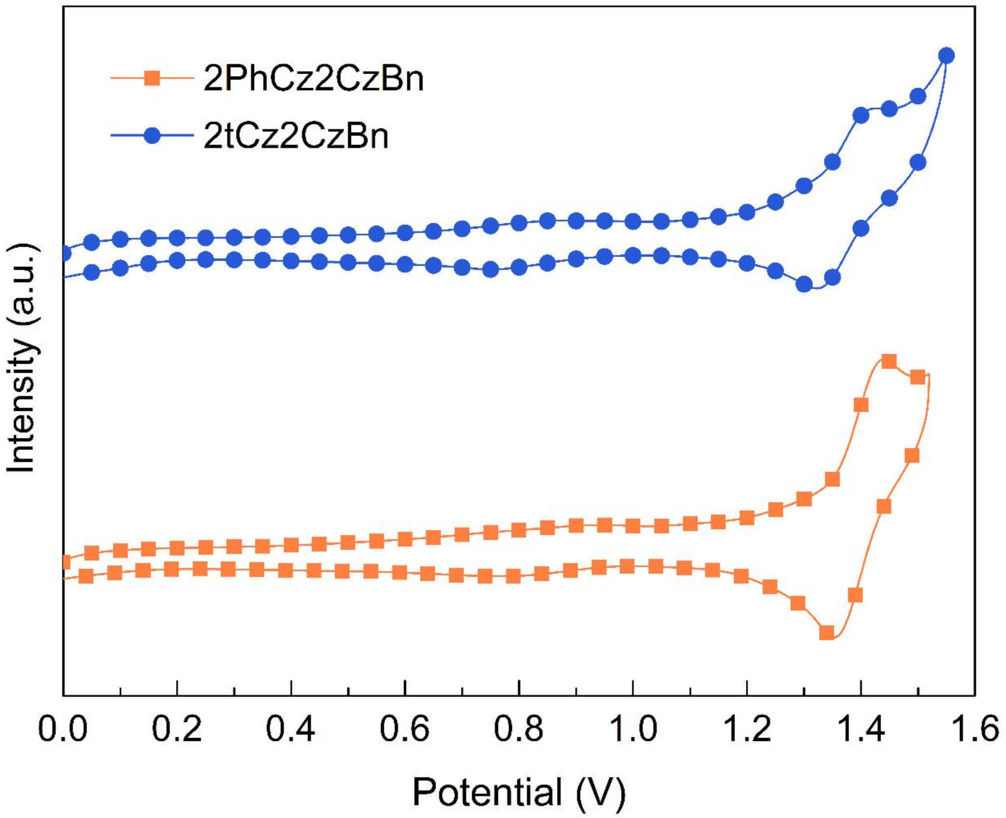

Figure S11. Cyclic voltammograms of 2PhCz2CzBn and 2tCz2CzBn in CH_2Cl_2 (10^{-5} M).

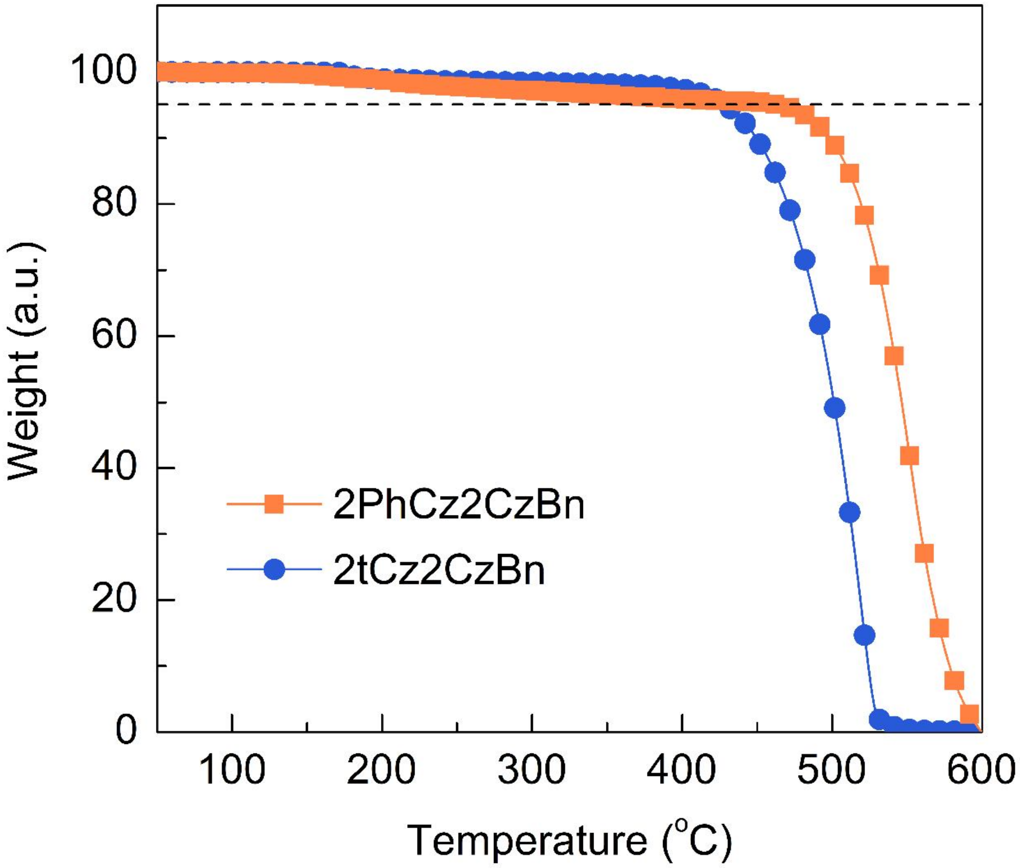

Figure S12. TGA curves of 2PhCz2CzBn and 2tCz2CzBn.

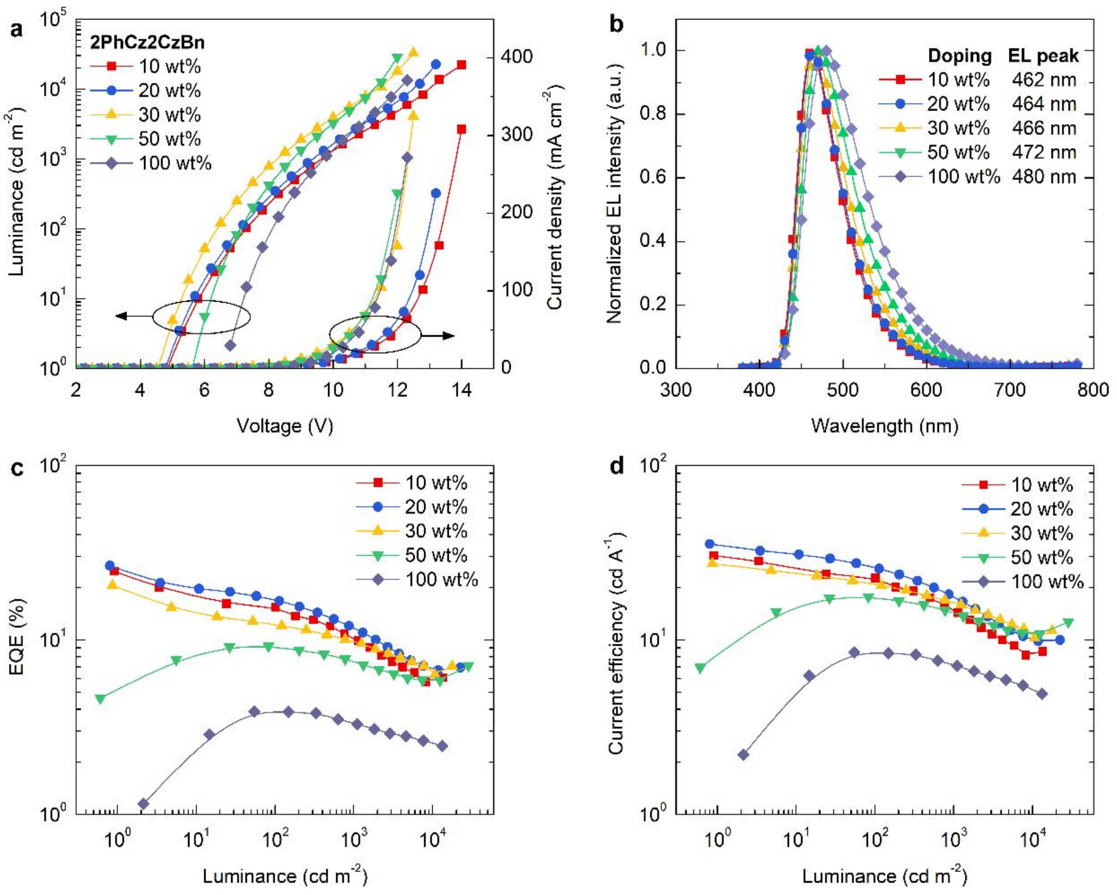

Figure S13. Performance characteristics of blue OLEDs with different 2PhCz2CzBn doping concentration. a) Current density-voltage-luminance (J-V-L) characteristics. b) Electroluminescence (EL) spectra at 1000 cd m^{-2}. c) External quantum efficiency (EQE) versus luminance. d) Current efficiency versus luminance.

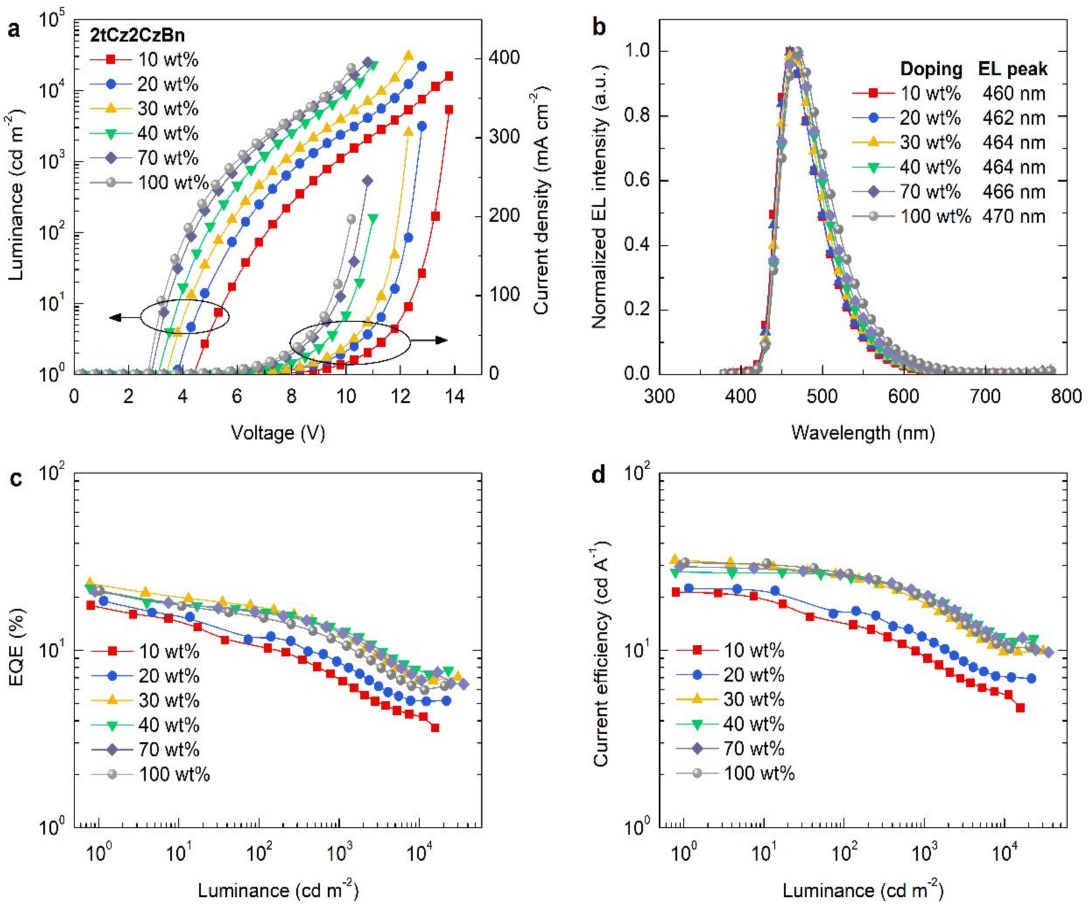

Figure S14. Performance characteristics of blue OLEDs with different 2tCz2CzBn doping concentration. a) Current density-voltage-luminance (J-V-L) characteristics. b) Electroluminescence (EL) spectra at 1000 cd m^{-2}. c) External quantum efficiency (EQE) versus luminance. d) Current efficiency versus luminance.

Table S4. Device performance of blue OLEDs based on different doping concentrations of 2PhCz2CzBN and 2tCz2CzBN.

Emitters	Doping concentration [wt%]	V_{on}[a] [V]	λ_{EL}[b] [nm]	CIE(x,y)[b]	EQE[c] [%]	CE[c] [cd A^{-1}]	L_{max}[d] [cd m^{-2}]
2PhCz2CzBn	10	4.8	462	(0.153,0.188)	24.7/15.4/10.3	30.3/22.6/14.9	13570
	20	4.7	464	(0.154,0.200)	26.6/17.2/11.7	35.4/26.1/17.3	22510
	30	4.5	466	(0.161,0.230)	20.5/12.2/9.8	27.3/20.9/16.3	32340
	50	5.6	472	(0.173,0.279)	9.2/9.0/7.4	17.6/17.3/14.3	28600
	100	6.7	480	(0.195,0.333)	3.9/3.9/3.3	8.5/8.5/7.1	13330
2tCz2CzBn	10	4.4	460	(0.151,0.170)	17.9/10.5/6.7	21.3/14.0/9.0	15850
	20	3.7	462	(0.152,0.176)	19.0/12.2/8.6	22.3/17.0/11.5	21800
	30	3.4	464	(0.153,0.193)	23.8/17.1/12.4	32.2/26.0/18.2	30290
	40	3.1	464	(0.155,0.210)	22.4/16.7/12.8	27.6/25.8/20.0	23020
	70	2.9	466	(0.158,0.220)	21.4/16.0/12.5	29.6/26.5/20.3	35510
	100	2.7	470	(0.167,0.248)	21.6/15.3/10.8	31.1/27.1/18.9	20590

[a] Turn-on voltage (V_{on}) at 1 cd m^{-2}. [b] EL peak wavelength and Commission Internationale de I'Eclairage (CIE) coordinates at 1000 cd m^{-2}. [c] External quantum efficiency (EQE) and current efficiency (CE) at their maximum, 100 cd m^{-2}, and 1000 cd m^{-2}. [d] Maximum luminance (L_{max}).

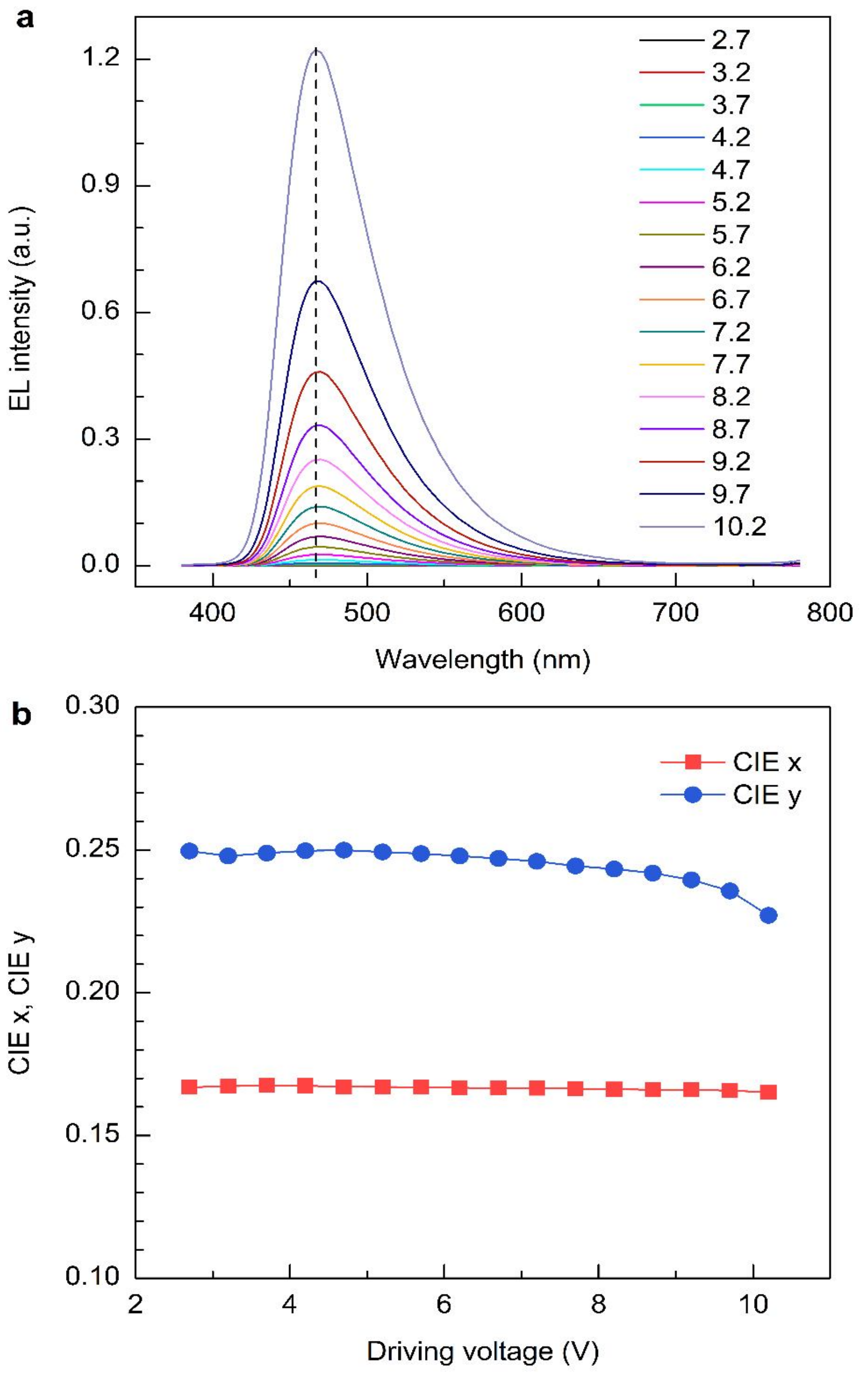

Figure S15. a) EL spectra of blue TADF OLED with non-doped 2tCz2CzBn emitter under various driving voltages. b) CIE color coordinates as a function of the driving voltage.

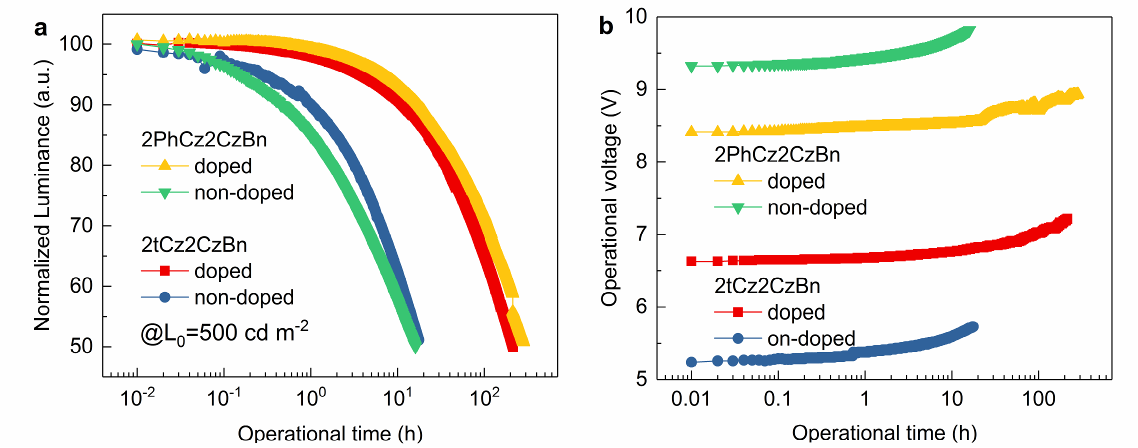

Figure S16. Operational lifetime measurements of doped and non-doped blue devices at an initial luminance of 500 cd m^{-2}. a) Normalized luminance versus operational time. b) The change of the driving voltage versus operational time.

Table S5. Comparison of the representative blue TADF emitters in doped and non-doped OLEDs reported in the literatures.

Emitters	Doping [wt%]	V_{on} [V]	EL peak [nm]	CIE (x, y)	EQE [%]	CE [cd A^{-1}]	L_{max} [cd m^{-2}]	Ref.
2tCz2CzBn	30	3.4	464	(0.15,0.19)	23.8	32.2	30290	This work
2tCz2CzBn	non-doped	2.7	470	(0.17,0.25)	21.6	31.1	20590	This work
DCBPy	5	2.8	488	(0.17,0.36)	24.0	54.7	10300	[1]
PXZ-BIP	10	2.82	497	(0.22,0.42)	21.0	51.6		[2]
MA-TA	10	5.1	465	(0.15,0.19)	22.1		<300	[3]
CzBPCN	5		460	(0.14,0.12)	14.0		<500	[4]
3Ph2CzCzBN	20	3.8	486	(0.18,0.39)	17.9	41.7	~40000	[5]
5CzBN	20			(0.19,0.41)	18.0	44.9		[6]
2a	6	~4.2	487	(0.19,0.35)	20.6			[7]
2b	6	~4.2	478	(0.17,0.27)	16.8			[7]
2c	6	~4.3	477	(0.18,0.28)	14.6			[7]
PAC	non-doped		458	(0.15,0.13)	10.48	12.37	16776	[8]
2SPAc-HPM	20	3.5	487	(0.18,0.34)	25.56	57.50	7200	[9]
2SPAc-MPM	20	3.6	479	(0.17,0.29)	24.34	50.00	4100	[9]
2SPAc-PPM	30	3.6	484	(0.18,0.32)	31.45	68.77	5600	[9]
MPAc-BS	50	2.8	503	(0.20,0.51)	25.3	72.1	12000	[10]
MPAC-BS	non-doped	3.4	487	(0.15,0.36)	22.8	49.9	5500	[10]
MPAC-BO	50	3.0	489	(0.16,0.38)	24.9	57.0	~4000	[10]
MPAC-BO	non-doped	3.5	474	(0.14,0.23)	21.3	33.6	1800	[10]
PXB-DI	20	3.5		(0.16,0.34)	37.4	66.2		[11]
PXB-mIC	20	3.5		(0.14,0.18)	18.8	22.1		[11]
p-AC-DBNA	10	3.3	488	(0.17,0.36)	20.5	47.4	40750	[12]
p-AC-DBNA	non-doped	2.7	516	(0.28,0.54)	14.1	47.1	27600	[12]
DCzBN4	10	3.5	468	(0.16,0.23)	18.0	24.2		[13]
SpiroAC-TRZ	12	2.6	490	(0.18,0.43)	36.7	94		[14]
SpiroAC-TRZ	non-doped	3.3		(0.19,0.42)	15.4	36.1	7383	[15]
TspiroS-TRZ	30	3.1	481	(0.17,0.33)	33.3	71.1		[15]
TspiroS-TRZ	non-doped	3.3	481	(0.16,0.30)	20.0	41.1	5119	[15]
DMA-THX	10	3.5	462	(0.14,0.14)	18.4	18.2	1460	[16]
mSOAD	36	3.6	480	(0.18,0.30)	16.6	34.6		[17]
mSOAD	non-doped	3.1	488	(0.18,0.32)	14.0	31.7		[17]
TDBA–Ac	20	3.8	465	(0.14,0.15)	25.71	27.73	9014	[18]
TDBA–DI	20	3.1	480	(0.15,0.28)	38.15	64.38	47,680	[18]
DMAc-DPS	10	3.7		(0.16,0.20)	19.8			[19]
DMAc-DPS	non-doped	4.1		(0.16,0.29)	19.5		5970	[20]

References

[1] P. Rajamalli, N. Senthilkumar, P. Gandeepan, P. Huang, M. Huang, C. Ren-Wu, C. Yang, M. Chiu, L. Chu, H. Lin, C. Cheng, A New Molecular Design Based on Thermally Activated Delayed Fluorescence for Highly Efficient Organic Light Emitting Diodes. *J. Am. Chem. Soc.* **2016**, *138*, 628.

[2] T. Ohsawa, H. Sasabe, T. Watanabe, K. Nakao, R. Komatsu, Y. Hayashi, Y. Hayasaka, J. Kido, A Series of Imidazo[1,2-f]phenanthridine-Based Sky-Blue TADF Emitters Realizing EQE of over 20%. *Adv. Opt. Mater.* **2019**, *7*, 1801282.

[3] Y. Wada, S. Kubo, H. Kaji, Adamantyl Substitution Strategy for Realizing Solution-Processable Thermally Stable Deep-Blue Thermally Activated Delayed Fluorescence Materials. *Adv. Mater.* **2018**, *30*, 1705641.

[4] Y. J. Cho, S. K. Jeon, S. Lee, E. Yu, J. Y. Lee, Donor Interlocked Molecular Design for Fluorescence-like Narrow Emission in Deep Blue Thermally Activated Delayed Fluorescent Emitters. *Chem. Mater.* **2016**, *28*, 5400.

[5] C. Chan, M. Tanaka, H. Nakanotani, C. Adachi, Efficient and Stable Sky-Blue Delayed Fluorescence Organic Light-Emitting Diodes with CIEy Below 0.4. *Nat. Commun.* **2018**, *9*, 5036.

[6] H. Noda, H. Nakanotani, C. Adachi, Excited State Engineering for Efficient Reverse Intersystem Crossing. *Sci. Adv.* **2018**, *4*, eaao6910.

[7] S. Hirata, Y. Sakai, K. Masui, H. Tanaka, S. Y. Lee, H. Nomura, N. Nakamura, M. Yasumatsu, H. Nakanotani, Q. Zhang, K. Shizu, H. Miyazaki, C. Adachi, Highly Efficient Blue Electroluminescence Based on Thermally Activated Delayed Fluorescence. *Nat. Mater.* **2015**, *14*, 330.

[8] Y. Xu, X. Liang, X. Zhou, P. Yuan, J. Zhou, C. Wang, B. Li, D. Hu, X. Qiao, X. Jiang, L. Liu, S. J. Su, D. Ma, Y. Ma, Highly Efficient Blue Fluorescent OLEDs Based on Upper Level Triplet-Singlet Intersystem Crossing. *Adv. Mater.* **2019**, *31*, 1807388.

[9] B. Li, Z. Li, T. Hu, Y. Zhang, Y. Wang, Y. Yi, F. Guo, L. Zhao, Highly Efficient Blue Organic Light-Emitting Diodes from Pyrimidine-Based Thermally Activated Delayed Fluorescence Emitters. *J. Mater. Chem. C* **2018**, *6*, 2351.

[10] I. S. Park, K. Matsuo, N. Aizawa, T. Yasuda, High-Performance Dibenzoheteraborin-Based Thermally Activated Delayed Fluorescence Emitters: Molecular Architectonics for Concurrently Achieving Narrowband Emission and Efficient Triplet-Singlet Spin Conversion. *Adv. Funct. Mater.* **2018**, *28*, 1802031.

[11] D. H. Ahn, H. Lee, S. W. Kim, D. Karthik, J. Lee, H. Jeong, J. Y. Lee, J. H. Kwon, Highly Twisted Donor-Acceptor Boron Emitter and High Triplet Host Material for Highly Efficient Blue Thermally Activated Delayed Fluorescent Device. *ACS App. Mat. & Int.* **2019**, *11*, 14909.

[12] G. Meng, X. Chen, X. Wang, N. Wang, T. Peng, S. Wang, Isomeric Bright Sky-Blue TADF Emitters Based on Bisacridine Decorated DBNA: Impact of Donor Locations on Luminescent and Electroluminescent Properties. *Adv. Opt. Mater.* **2019**, *7*, 1900130.

[13] C. Y. Chan, L. S. Cui, J. U. Kim, H. Nakanotani, C. Adachi, Rational Molecular Design for Deep-Blue Thermally Activated Delayed Fluorescence Emitters. *Adv. Funct. Mater.* **2018**, *28*, 1706023.

[14] T. Lin, T. Chatterjee, W. Tsai, W. Lee, M. Wu, M. Jiao, K. Pan, C. Yi, C. Chung, K. Wong, C. Wu, Sky-Blue Organic Light Emitting Diode with 37% External Quantum Efficiency Using Thermally Activated Delayed Fluorescence from Spiroacridine-Triazine Hybrid. *Adv. Mater.* **2016**, *28*, 6976.

[15] W. Li, B. Li, X. Cai, L. Gan, Z. Xu, W. Li, K. Liu, D. Chen, S. Su, Tri-Spiral Donor Strategy for High Efficiency and Versatile Blue TADF Materials. *Angew. Chem., Int. Ed.* **2019**. https://doi.org/10.1002/anie.201904272.

[16] Y. P. Jeon, B. K. Kong, E. J. Lee, K. Yoo, T. W. Kim, Ultrahighly-Efficient and Pure Deep-Blue Thermally Activated Delayed Fluorescence Organic Light-Emitting Devices Based on Dimethylacridine/Thioxanthene-S,S-Dioxide. *Nano Energy* **2019**, *59*, 560.

[17] J. Li, R. Zhang, Z. Wang, B. Zhao, J. Xie, F. Zhang, H. Wang, K. Guo, Zig-Zag Acridine/Sulfone Derivative with Aggregation-Induced Emission and Enhanced Thermally Activated Delayed Fluorescence in Amorphous Phase for Highly Efficient Nondoped Blue Organic Light-Emitting Diodes. *Adv. Opt. Mater.* **2018**, *6*, 1701256.

[18] D. H. Ahn, S. W. Kim, H. Lee, I. J. Ko, D. Karthik, J. Y. Lee, J. H. Kwon, Highly Efficient Blue Thermally Activated Delayed Fluorescence Emitters Based on Symmetrical and Rigid Oxygen-Bridged Boron Acceptors. *Nat. Photon.* **2019**, *13*, 540.

[19] Q. Zhang, B. Li, S. Huang, H. Nomura, H. Tanaka, C. Adachi, Efficient Blue Organic Light-Emitting Diodes Employing Thermally Activated Delayed Fluorescence. *Nat. Photon*. **2014**, *8*, 326.

[20] Q. Zhang, D. Tsang, H. Kuwabara, Y. Hatae, B. Li, T. Takahashi, S. Y. Lee, T. Yasuda, C. Adachi, Nearly 100% Internal Quantum Efficiency in Undoped Electroluminescent Devices Employing Pure Organic Emitters. *Adv. Mater.* **2015**, *27*, 2096.
